# An integrative transcriptome analysis framework for drug efficacy and similarity reveals drug-specific signatures of anti-TNF treatment in a mouse model of inflammatory polyarthritis

**DOI:** 10.1371/journal.pcbi.1006933

**Published:** 2019-05-09

**Authors:** Niki Karagianni, Ksanthi Kranidioti, Nikolaos Fikas, Maria Tsochatzidou, Panagiotis Chouvardas, Maria C. Denis, George Kollias, Christoforos Nikolaou

**Affiliations:** 1 Biomedcode Hellas SA, Vari, Greece; 2 Department of Biology, University of Crete, Heraklion, Greece; 3 Institute of Immunology, Biomedical Sciences Research Center (BSRC), ‘Alexander Fleming’, Vari, Greece; 4 Department of Physiology, School of Medicine, National Kapodistrian University, Athens, Greece; 5 Institute of Molecular Biology and Biotechnology (IMBB), Foundation of Research and Technology (FORTH), Heraklion, Greece; University of Cambridge, UNITED KINGDOM

## Abstract

Anti-TNF agents have been in the first line of treatment of various inflammatory diseases such as Rheumatoid Arthritis and Crohn’s Disease, with a number of different biologics being currently in use. A detailed analysis of their effect at transcriptome level has nevertheless been lacking. We herein present a concise analysis of an extended transcriptomics profiling of four different anti-TNF biologics upon treatment of the established hTNFTg (Tg197) mouse model of spontaneous inflammatory polyarthritis. We implement a series of computational analyses that include clustering of differentially expressed genes, functional analysis and random forest classification. Taking advantage of our detailed sample structure, we devise metrics of treatment efficiency that take into account changes in gene expression compared to both the healthy and the diseased state. Our results suggest considerable variability in the capacity of different biologics to modulate gene expression that can be attributed to treatment-specific functional pathways and differential preferences to restore over- or under-expressed genes. Early intervention appears to manage inflammation in a more efficient way but is accompanied by increased effects on a number of genes that are seemingly unrelated to the disease. Administration at an early stage is also lacking in capacity to restore healthy expression levels of under-expressed genes. We record quantifiable differences among anti-TNF biologics in their efficiency to modulate over-expressed genes related to immune and inflammatory pathways. More importantly, we find a subset of the tested substances to have quantitative advantages in addressing deregulation of under-expressed genes involved in pathways related to known RA comorbidities. Our study shows the potential of transcriptomic analyses to identify comprehensive and distinct treatment-specific gene signatures combining disease-related and unrelated genes and proposes a generalized framework for the assessment of drug efficacy, the search of biosimilars and the evaluation of the efficacy of TNF small molecule inhibitors.

## Author summary

A number of anti-TNF drugs are being used in the treatment of inflammatory autoimmune diseases, such as Rheumatoid Arthritis and Crohn’s Disease. Despite their wide use there has been, to date, no detailed analysis of their effect on the affected tissues at a transcriptome level. In this work we applied four different anti-TNF drugs on an established mouse model of inflammatory polyarthritis and collected a large number of independent biological replicates from the synovial tissue of healthy, diseased and treated animals. We then applied a series of bioinformatics analyses in order to define the sets of genes, biological pathways and functions that are affected in the diseased animals and modulated by each of the different treatments. Our dataset allowed us to focus on previously overlooked aspects of gene regulation. We found that the majority of differentially expressed genes in disease are under-expressed and that they are also associated with functions related to Rheumatoid Arthritis comorbidities such as cardiovascular disease. We were also able to define gene and pathway subsets that are not changed in the disease but are, nonetheless, altered under various treatments and to use these subsets in drug classification and assessment. Through the application of machine learning approaches we created quantitative efficiency profiles for the tested drugs, which showed some to be more efficiently addressing changes in the inflammatory pathways, while others being quantitatively superior in restoring gene expression changes associated to disease comorbidities. We thus, propose a concise computational pipeline that may be used in the assessment of drug efficacy and biosimilarity and which may form the basis of evaluation protocols for small molecule TNF inhibitors.

## Introduction

In an era of unprecedented accumulation of biomedical data, our understanding of the mechanisms of the development of complex diseases is greatly enabled by the performance of high-throughput experiments and their subsequent analyses at various levels that range from single genes to biological pathways, modules and networks [1, 2]. Genome-wide transcriptomic profiling has been instrumental in providing accurate representations of the expression programs in homeostasis and disease as well as before and after pharmaceutical interventions [3–7]. Among various pathological conditions, inflammatory diseases such as Rheumatoid Arthritis (RA) present great challenges in the understanding of the process through which an initial trigger may lead to generalized and highly variable changes at molecular, cellular and eventually organ level [8]. In this respect, animal models have proven invaluable in the detailed study of these intricate mechanisms and have been the choice of preference in many studies due to particular advantages such as accessibility of material, robustness and standardization [9,10].

Recently, an attempt to assess the differential properties of different substances used in the treatment of RA was performed at whole blood transcriptome level of human patients [11], producing different gene signatures that reflected the mechanistic differences of the tested biologics (an anti-TNF, an anti IL6-R and an inhibitor of T-cell maturation). Among the various therapeutic agents used in the treatment of RA, anti-TNF antibodies have been the primary line of defense since TNF was shown to be a major driver of the disease [12]. In this context, a concise analysis of the effect of the different anti-TNF biologics at transcriptome level has been lacking. The need for investigating the variability in differential patterns of anti-TNF agents [13] has been supported by findings at various levels that include the stratification of RA subtypes [14], cell-type dependent responses [15] and variability among cells of the same type, namely fibroblast-like synoviocytes [16,17].

In this work, we present a computational analysis [18–24] based on a standardized and highly robust protocol involving the administration of four different anti-TNF agents in the hTNFTg (Tg197) humanized mouse model of RA, that has been essential in proving the central role of TNF in the arthritis pathology and its validity as a major therapeutic target [12]. The large number of analyzed profiles and the incorporation of both untreated and healthy samples in our study, enables us to go beyond a simple recording of differentially expressed genes and enriched pathways, which are the usual output of such analyses. Instead, we present a framework for the assessment of drug similarity at various functional levels through the implementation of a combination of gene clustering and state of the art classification methods. Treatment profiles were analyzed at two levels, focusing first on genes that are associated to the disease and then on those that are altered by the treatment even if unchanged in transgenic (diseased) animals. In this way we are able to pinpoint a number of functional attributes that are treatment-specific and accordingly devise measures of profile similarity between treatments and the healthy state. Our results suggest subtle, yet significant differences between the different biologics as well as between different intervention timing (early or late).

Our work provides a general framework for the comparison of treatment-specific transcriptomes that may assist in a) the detailed profiling of the gene and functional modules addressed (or left unaffected) by a given treatment b) the multi-level assessment of the treatment’s efficiency in restoring gene expression levels and c) similarity searches between different pharmaceutical agents. Implementation of our analyses may thus lead to interesting applications in the search of biosimilarity and specify suggestions in the evaluation of small molecule inhibitors.

## Results

### In vivo experiments

**Fig 1:**
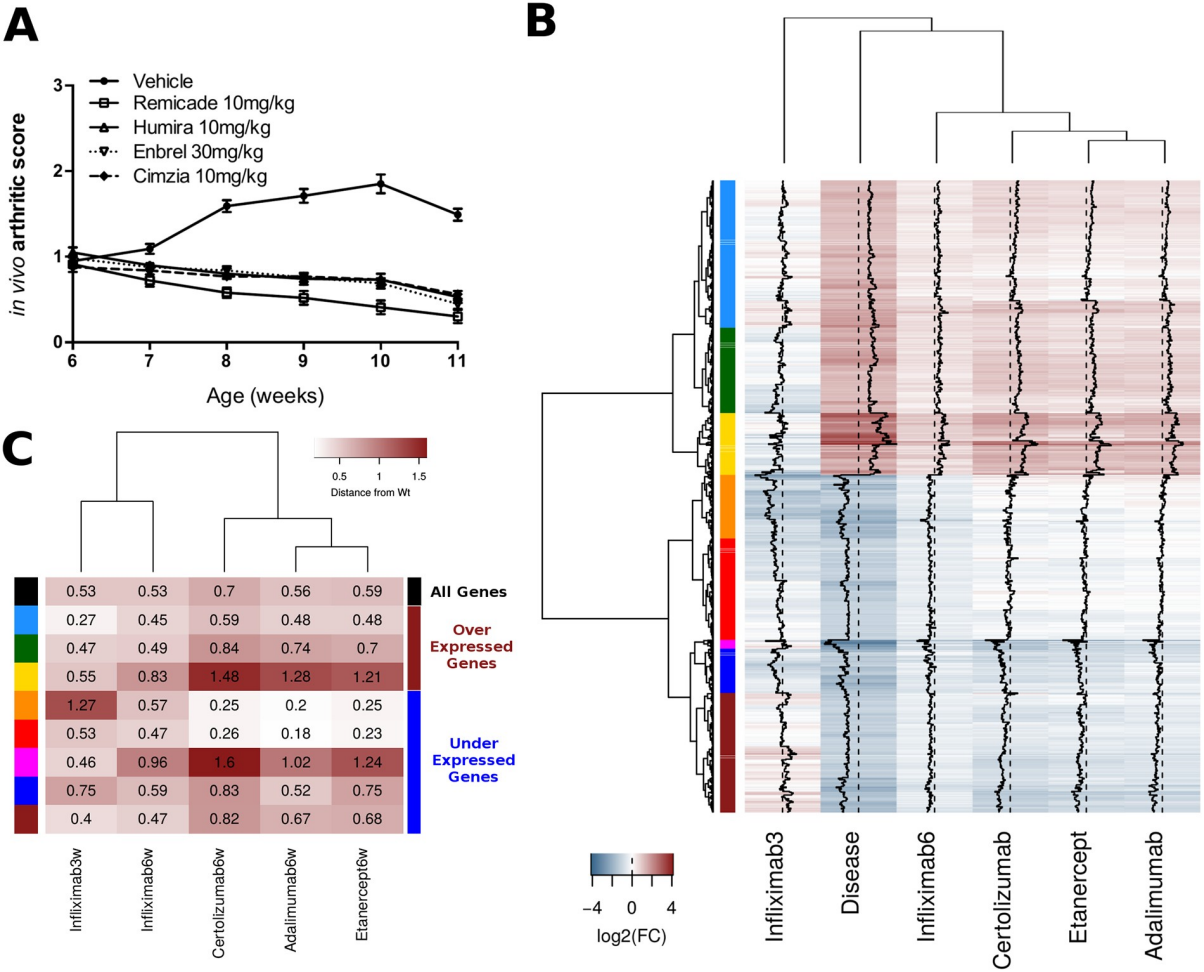
Clustering analysis of disease-associated genes. A. In vivo arthritic score depicting the time course progression of the arthritis pathology in hTNFTg mice and the response to treatment with anti-hTNF biologics. Treatment at a therapeutic stage started at 6 weeks with groups of mice of the same age (gender balanced) receiving either saline, infliximab (Remicade, Janssen Biotech), certolizumab pegol (Cimzia, UCB) or adalimumab (Humira, Abbvie) administered at 10 mg/kg intraperitoneally twice weekly or etanercept (Enbrel, Pfizer) administered subcutaneously at 30 mg/kg thrice weekly. During a treatment period of 6 weeks mice were regularly monitored and scored for the progress of the disease pathology and for their overall health status. Intervention at an earlier stage (3 weeks) was performed with the same dose of infliximab (Remicade, Janssen Biotech). B. Differential gene expression levels against wild-type (WT) samples for 867 disease-associated differentially expressed genes (|log2FC|> = 1 and p-value< = 0.05). The coloured bar on the left side of the heatmap correspond to 8 gene clusters on the basis of their expression levels across all conditions (disease and treatments). Black dashed line corresponds to no change, black full line is the trendline corresponding to differential expression levels. C. Aggregative distance from WT (see Materials and methods for details) for each of the 8 disease-associated gene clusters and for each of the 5 treatment regimens. Cluster characterization is based on comparative observations of gene expression levels shown in A (see also S1 Fig). “Over” and “under” indicate over- and under-expression. See text for details.

The hTNFTg (Tg197) human TNF transgenic model develops chronic inflammatory polyarthritis with 100% incidence and with clinical manifestations and histological findings very similar to those of the human disease [12] becoming evident as early as 3 weeks of age with signs progressively worsening as animals age. Animals were treated in a therapeutic regimen from week 6 of age when pathology is already established or in a prophylactic regimen from week 3 of age when pathology is at an early stage. Groups of mice of the same age (gender balanced) received either saline or one of the following anti-TNF agents: a) the chimeric monoclonal antibody infliximab (Remicade, Janssen Biotech), b) the pegylated antigen binding fragment (Fab) certolizumab pegol (Cimzia, UCB) c) he fully human monoclonal antibody adalimumab (Humira, Abbvie) and d) the fusion protein TNFR2/IGg1Fc etanercept (Enbrel, Pfizer). The first three were administered at 10 mg/kg intraperitoneally twice weekly, while etanercept was administered subcutaneously at 30 mg/kg thrice weekly. Apart from a standard therapeutic stage administration, infliximab was also administered with the same dosage but starting at an earlier stage (3 weeks). During a treatment period of 6 weeks mice were regularly monitored and scored for the progress of the disease pathology and for their overall health status. All therapeutic treatments showed very similar disease progression patterns with an overall in vivo arthritic score dropping steadily from the onset of the intervention, suggesting similar patterns of remission of the hTNFTg arthritis pathology (Fig 1A).

### Patterns of differentially expressed genes

Sets of Differentially Expressed (DE) genes were defined on the basis of standard thresholds i.e. a |log_2_FC|≥1 combined with an adjusted p-value≤0.05, against either wild-type (WT) or transgenic (TG) samples. The cutoffs used, corresponded to values lying beyond two standard deviations (+/-2σ) in a symmetrical log_2_FC distribution and resulted in the selection of <5% of the total genes. We then combined DE genes in various lists depending on the initial comparison undertaken and focused our analyses on the following DE gene subsets:

1. 867 DE genes resulting from the comparison of TG vs WT and defining an arthritis pathology associated profile.
2. 1064 DE genes resulting from the comparison of all conditions with WT and thus including, apart from the arthritis profile (867), all genes differentiated from WT with at least one treatment (197).
3. 1338 DE genes resulting from the union of genes differentiated either from TG or from WT with at least one treatment. This set includes the arthritis profile (867), plus the genes differentiated from WT or TG upon any single treatment (471).

For the initial part of our analysis we focused on the first subset of the 867 TG/WT DE genes, which was used as a disease-associated gene set that allowed us to monitor the functions and pathways that are mainly affected in the diseased animals regardless of pharmaceutical intervention. The more extensive sets of 1064 and 1338 DE genes were used in further analyses of treatment-specific properties, since they included genes whose expression was modulated by the treatment even if they remained unchanged in the diseased animals. They thus represented more inclusive lists comprising genes that are not directly related to the diseased state.

### Gene expression changes of disease-associated genes

Expression patterns of the 867 disease-associated genes in treated and untreated hTNFTg against wild-type samples are shown in Fig 1A. One can see that the DE genes are roughly equally divided in over- (404) and under-expressed genes (463) in the diseased versus wild-type state. The slight predominance of under-expressed genes is a distinguishing feature of our analysis compared to previous attempts on human samples, where the lack of normal controls often shifts the balance to over-expressed genes in ratios that vary from 3:1 to 6:1 [25–27]. The large number of under-expressed genes points towards a number of functions that are associated to the diseased state but are not necessarily linked to known, implicated immune and inflammatory pathways.

Focusing on particular treatment profiles, we found that the prophylactic intervention with infliximab was the one that most readily restored expression to wild-type levels. This was expected due to the time of intervention at 3 weeks of age and prior to full disease development. On the other hand, in this particular intervention, gene expression changes were in many cases over-compensated, leading to expression levels being reversed, that is genes over-expressed in diseased mice, being under-expressed in treated samples and vice versa.

Among the therapeutic interventions, infliximab appears to be the treatment with more direct effects on over-expressed genes, while adalimumab, etanercept and certolizumab pegol present great similarity in terms of expression levels, showing increased restoring potential for a large set of under-expressed genes. Differences between infliximab and the rest of the treatments were primarily quantitative, an observation that is in accordance with a mild advantage of infliximab in the clinical data (see Fig 1A). For specific subsets of genes, restoration to normal (wild-type) levels was more extensive with infliximab compared to the other three substances.

Based on the expression patterns of treated and untreated mice, we clustered the 867 disease-associated genes in 8 distinct clusters, a number that was indicated by the application of a Silhouette consistency test (see Materials and methods). Of the 8 clusters, 5 contained genes that were under-expressed in disease (463 genes), while 3 included a total number of 404 over-expressed genes, as may be seen in the coloured side bar of Fig 1B. A more detailed inspection of the differential expression values for each cluster from Fig 1B reveals different patterns of response for the different treatments.

Among the three over-expressed gene clusters, the first (light blue) shows all treatments sufficient to bring expression down to wild-type levels. The second cluster (dark green) contains genes for which prophylactic intervention drives a partial reversal of gene expression levels, while therapeutic treatment is more or less comparable regardless of the administered biologic, with a slight but quantifiable advantage for infliximab. The third cluster (yellow) comprises the subset of the most over-expressed disease-associated genes (compare with Disease state in Fig 1B) which are, expectedly, partially restored to normal levels by therapeutic interventions but are, interestingly, strongly reversed in the prophylactic one (see also S1 Fig). This reversal may indicate that the subset of the most over-expressed genes is probably not present, or at least not fully formed before later stages of the disease. Thus, it contains genes, which are reversed when the treatment is administered early but not fully addressed at a later stage, an observation which may bear significant implications on the dynamics of the disease.

Under-expressed genes are clustered in five groups of variable size. Going from top to bottom (Fig 1B), for two of the five (Fig 1B, shown in orange and red) there is a quantitative difference in gene expression restoring potential between adalimumab, etanercept and certolizumab pegol against infliximab. What is also worth mentioning, is that for these clusters infliximab shows limited capacity to modulate gene expression at prophylactic intervention. This may be indicative of different expression dynamics between over- and under-expressed genes, with the first being deregulated earlier and thus addressed at a prophylactic stage while the latter being associated with a more delayed onset. A third under-expressed gene cluster (dark blue) comprises 61 genes for which gene expression is partially restored with all treatments, while a fourth, shown in brown at the bottom of Fig 1B shows partial resetting of gene expression levels with therapeutic interventions and a reversal (from under- to over-expressed) in the case of the prophylactic infliximab treatment. Lastly, a small cluster of only 12 genes (Fig 1B pink), contains the genes with the most acute under-expression patterns, which seem to be preferentially addressed by infliximab at prophylactic intervention but not by the rest of the treatments. The small number of these genes (12) does not allow for an enrichment analysis but in general they correspond to genes associated with collagen and myofibrils (Col10a1, Glt25d2, Xirp2, Myl2 and Clec3a) innate immunity (C7, Cytl1, Vsig4), the phospholpases Pla1a and Pla2g2a, the serine peptidase Htra4 and the developmental protein Hhip (heghehog interacting protein).

### Functional analysis of disease-associated genes

**Fig 2:**
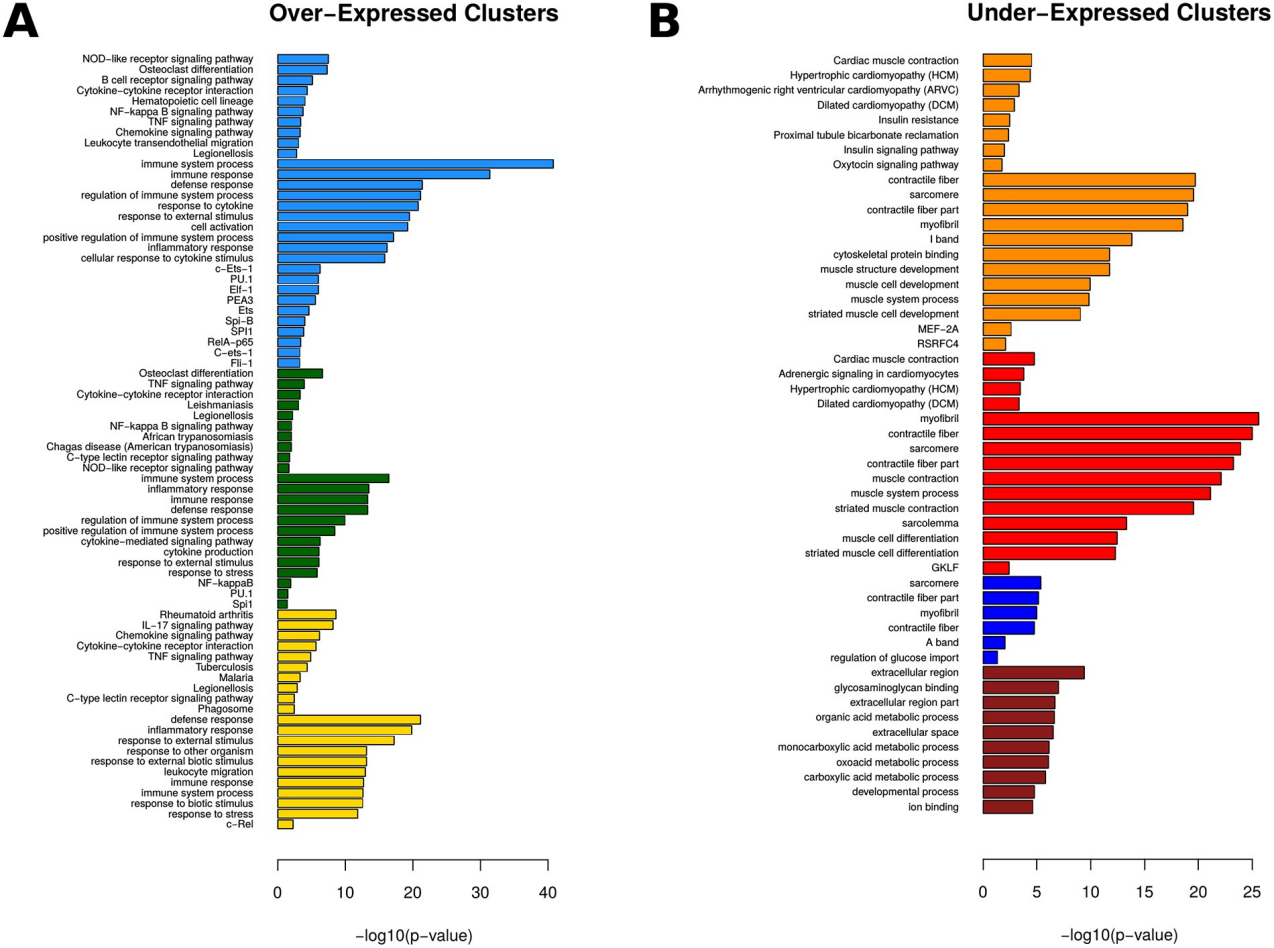
Functional analysis of disease-associated genes. Functional analysis summary for 7 out of 8 disease-associated gene clusters. The top functional terms from Gene Ontology (GO), KEGG Pathways and Transcription Factor binding sites (TRANSFAC) are reported separately for (A) over- and (B) under-expressed (right) gene clusters. Values are reported as -log10(p-value) of enrichment on the basis of a hypergeometric test, adjusted for an FDR of 95%. gProfiler was used for all levels of functional enrichment analysis.

In order to gain insight on the functional characteristics of the underlying genes we performed a functional enrichment analysis individually for each cluster. Functional enrichments were calculated with the implementation of gProfiler [20] as described in Methods and a summary of the most enriched terms at the levels of Gene Ontology, KEGG pathways and Transcription Factor targets is shown in Fig 2A and 2B.

Two main observations stem from the enrichments of over- and under-expressed gene clusters respectively. On one hand, terms related to the inflammatory and immune responses, including cytokine and chemokine signaling as well as infectious pathways and the associated transcription factors are prominent in all three over-expressed gene clusters (Fig 2A). On the other hand, there is a characteristic over-representation of functional terms related to muscle and heart functions and associated diseases, as well as metabolic and developmental pathways among the under-expressed genes (Fig 2B).

Heart failure and related cardiovascular diseases are known comorbidities of Rheumatoid Arthritis patients [28–30], while we recently showed this to be the case for the animal model under study [31] It is thus interesting to see that associated functions are linked with the under-expression of certain genes, which, as noted above, have been largely overlooked in human studies. In this respect, the aforementioned differential capacity of the analyzed anti-TNF agents to effectively restore under-expressed genes, could lead to more detailed insights on treatment-specific efficiency regarding indirect and secondary effects of the disease.

### Efficiency of treatment as similarity to wild-type gene expression levels

As a next step we wanted to assess the level of potential for gene expression resetting that may be achieved by each treatment. For this we employed a distance-from-WT calculation (see Materials and methods) for all disease-associated genes and for each of the gene expression clusters defined above. Mean absolute log_2_FC values were calculated for each cluster and for each treatment and the distance from WT was then used as an indication of treatment efficiency. The results are summarized in Fig 1C, where distances from WT are shown in the form of a clustered heatmap.

Overall mean distances (all genes, black) show small differences between treatments with infliximab lying closer to the healthy state, regardless of time of intervention. When looking into separate clusters one can observe that clusters associated with over-expressed genes, and with immune and inflammatory responses, respond better to infliximab at prophylactic stage. For the same over-expressed clusters, infliximab seems to have a small quantitative advantage at the therapeutic stage, over the rest of the treatments. On the other hand, adalimumab in particular but also etanercept and certolizumab pegol, are more readily restoring gene expression levels of under-expressed gene clusters, an effect that is especially strong for two of the largest clusters (orange and red) which are functionally associated with heart-related functions and diseases (see Fig 2B).

This similarity of the three agents is also reflected in the treatment clustering, in which they are placed together in a group that is separate from inflixιmab. It thus seems that one particular property discriminating infliximab from the rest of the other three anti-TNF agents is related to its increased efficiency in modulating the expression of inflammatory and immune-related genes that are up-regulated in disease. On the other hand, adalimumab, etanercept and certolizumab pegol show greater capacity in restoring gene expression levels for under-expressed genes associated with RA comorbidities.

### Total differentially expressed genes

When assessing pharmaceutical interventions, one of the main aspects we need to address is general changes that are not directly associated with the condition under treatment. In the context of our transcriptomic analyses this meant looking into the genes, whose expression changed between treatment and wild-type profiles but which were not altered in the diseased state, as well as into genes that were changed between treatment and diseased samples in general. In this sense, we may divide differentially expressed genes in three groups:

1. Genes whose expression changes in disease and is **restored** after treatment. This is the set of genes upon which the treatment exerts its main effect.
2. Genes whose expression changes in disease but is **not restored** after treatment. This is the set of genes for which treatment fails to stimulate a response or does so incompletely.
3. Genes whose expression does not change in disease but is nonetheless **altered** upon treatment. These are genes upon which treatment exerts a secondary effect, that is not directly associated to the disease.

Analysis of the corresponding genes belonging to each category resulted in a total of 1338 genes that were unequally distributed among treatments (S2 Fig). At a first-level quantitative assessment based on the number of genes, infliximab performs better in terms of restoration to wild-type levels, at both prophylactic and therapeutic interventions, with the numbers of non-restored genes being only 3 and 25 (out of a total of 867) respectively, compared to significantly higher numbers of non-restored genes for adalimumab (92), etanercept (106) and certolizumab pegol (175). When assessing the treatments through their distance from WT for this extended set of genes, it is infliximab at therapeutic intervention which now shows the highest efficiency (mean distance = 0.47) followed by the same substance when administered at prophylactic stage (mean distance = 0.53). Among the three remaining biologics differences are becoming clearer with adalimumab performing considerably better (mean distance = 0.61 compared to etanercept’s 0.72 and certolizumab’s 0.78) (S3 and S4 Figs). It may be noted here that, variability in human patients’ response to adalimumab treatment has been attributed to differential levels of cytokine expression in the synovium [32], which may reflect the quantitative effect compared to infliximab that we are observing.

At this first, purely quantitative level, our results are indicative of a more direct response at gene expression level for infliximab (particularly at prophylactic intervention) and adalimumab compared to etanercept and certolizumab pegol. When one looks at the altered genes, a time effect appears to become important, with infliximab at prophylactic intervention showing a greater number of genes (170) which is comparative to certolizumab (175) and etanercept (217), the latter being the therapeutic treatment with the highest number of non-disease associated modulated genes. Indeed, the time of intervention appears to be crucial since the same substance (infliximab) when administered at a later stage (6w) has practically no altered genes, suggesting maximal specificity. Overall, infliximab and adalimumab at a therapeutic stage of intervention show the greater number of restored genes combined with a small number of altered genes and thus appear, at this level, to reflect the most effective gene expression modulation patterns.

### Expression profiles of total differentially expressed genes

**Fig 3:**
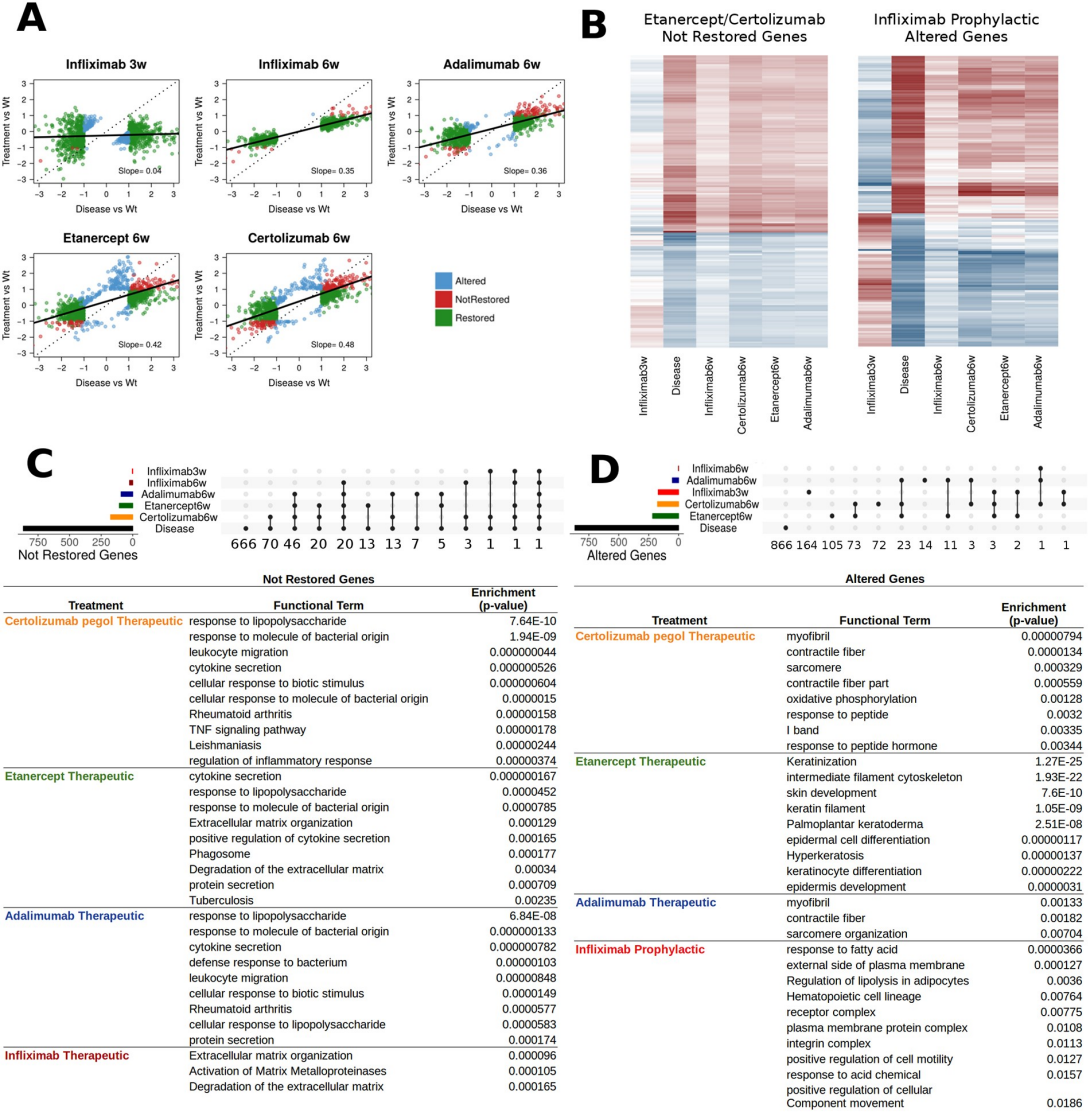
Assessing treatment differences on the basis of total differentially expressed genes. **A**. Differential expression levels against wild-type and transgenic profiles for DE genes. Green: disease-associated genes that are restored by treatment, Red: disease-associated genes that are not restored by treatment, Blue: genes that are altered by treatment but are not differentially expressed in disease. Dotted diagonal lines correspond to total lack of restoration. Full trendlines correspond to linear regression slopes between treatment and disease profiles for the set of studied genes. Slope values are shown inside each plot. B. Gene expression levels for two specific subsets of genes. Left: genes not restored by Etanercept and Certolizumab. Right: Genes altered by Infliximab at prophylactic intervention. Color code is the same as in Fig 1A (red: over-expressed, blue: under-expressed). C. Multiple gene intersection representations for not restored genes among the various treatments and the diseased state, performed with UpSetR (see Materials and methods). Horizontal represent total number of not restored genes in each treatment. Vertical bars correspond to numbers of genes occurring in the grid of treatments designated by the points underneath, where black dots represent the intersection tested (see Results for details). Top enriched functional analysis terms are referred to for each treatment separately. Functional analysis was performed with gProfiler as described in the text (see also Fig 1B). D. Multiple gene intersection representations for altered genes among the various treatments and the diseased state. Functional analysis table as in B. Treatments not present in the functional analysis had no enriched term due to very small number of genes.

In order to gain more detailed insight on how the response to treatment may be compared to the diseased state, we plotted gene expression values for the three gene categories (Restored: green, not Restored: red, Altered: blue) in two dimensional scatterplots shown in Fig 3A. Here, the expression of each gene in disease is shown on the horizontal axis and the corresponding value for the treated samples is shown on the vertical one. In this respect, a good overall response will have points distributed on a horizontal line around 0 (no change compared to wild-type), while points that fall away from the horizontal baseline will represent genes that are either non-responsive to treatment (red) or genes that are altered (blue). The diagonal dashed line corresponds to identical values in treatment and disease, therefore linear trendlines, based on the total number of genes, are representative of the overall response profile of the treatment.

Slopes greatly deviate from 1 (diagonal), with infliximab again showing the smallest slope values, closer to the desired horizontal. Infliximab at prophylactic intervention shows the smallest slope value, but this is mostly due to the small deviation of the altered genes. A close inspection of Fig 2A shows that altered genes (blue) for infliximab at prophylactic intervention fall on the opposite side of the trendline, which is indicative of gene expression reversals. One may observe this in Fig 3B, where (on the right) the gene expression values for the genes altered in this particular treatment show a strong reversal of expression levels against the diseased animals. Thus, a significant number of genes seem to be affected in a very acute way when intervention takes place early, as may be seen with a direct comparison to the infliximab therapeutic treatment (Fig 3B, right).

### Qualitative analysis of not-restored and altered gene overlaps between treatments

We next performed a detailed analysis of partial overlaps of not restored (Fig 3C) and altered genes (Fig 3D). Genes not restored to normal levels do not appear to be treatment-specific, as may be seen in extensive overlaps among all treatments (Fig 3C, layer of black dots spanning most conditions). Certolizumab pegol and etanercept in particular, share the highest number of not restored genes (88 genes in total, 20 of which are specific between them). Infliximab has 25 genes whose levels are not restored, 21 of which are shared with adalimumab. As may be seen in Fig 3A, these genes (red dots) are primarily over-expressed in disease and even though their deregulation is ameliorated upon treatment they remain quantitatively active, above the thresholds that qualify them as differentially expressed. This quantitative effect may explain the functional enrichments of not restored genes shown in the table accompanying Fig 3C. With the exception of infliximab, whose 25 not restored genes are enriched in functions related to the extracellular matrix and its degradation, the other three treatments show enrichments in immune, inflammatory and infectious pathways (see also S5 Fig). Even though this seems, at first, counter-intuitive it is supported by the results in Fig 3A and 3B where one can see that there is a quantitative lag in restoring the expression of disease-associated genes. Thus, even though treatments may show similar macroscopic effects (Fig 1A), they have quantifiable differences at the molecular level (S4 Fig), an observation that highlights the importance of -omics approaches in the analysis of detailed treatment profiles.

An identical gene overlap and functional analysis was carried out for altered genes (Fig 3D). In the case of altered genes, effects appear to be largely treatment-specific, with most of the altered genes belonging to particular treatments (compare layers of dots in 3C and 3D). As noted earlier, the most striking observation is the significant number of altered genes in the case of infliximab prophylactic intervention (170). Of these, 164 are exclusive to the particular treatment, which is indicative that they are largely associated to the age and the different dynamics of gene expression of younger mice that have not yet manifested the disease in its full proportions. These differences are also reflected in the functional analysis where terms related to development, cell motility and fatty acid and lipid metabolism are the ones primarily enriched. What is particularly interesting for altered genes of the infliximab prophylactic treatment, is their expression levels, which show a strong reversal compared to the disease state (Fig 3B, right). This is yet another indication of the strong effects of the prophylactic intervention, effects that extend beyond the disease-associated gene set.

Altered genes of adalimumab, etanercept and certolizumab are also largely treatment-specific (Fig 3B, right). Etanercept and certolizumab share a significant percentage due to the overall large numbers of altered genes (217 and 175 respectively). At the functional level, however, differences are also apparent, with certolizumab’s genes being enriched in structural proteins and oxidative metabolism, etanercept’s being very strongly associated with skin functions and differentiation and adalimumab having only a handful of enriched pathways related to structural proteins.

Together, the analysis of not restored and altered genes reveals differences at both qualitative (gene lists and functions) and quantitative (extent or reversal of gene expression levels). Therefore, accurate representations of treatment regimens at transcriptomic level require the analysis of such inclusive gene sets.

### Classification of interventions with Random Forests

We set out to build a classification model that would preferably discriminate between the treatments and the diseased samples, while at the same time, provide us with insight on the most important genes and pathways. We chose a Random Forest approach since it combines both such characteristics and implemented it on the total number of profiles, including wild-type and transgenic [24]. The best out of 1000 models (see Materials and methods) gave a perfect discrimination between wild-type and transgenic profiles (which was expected given their considerable differences) but also led to a significant improvement in the classification of the different treatments.

**Fig 4:**
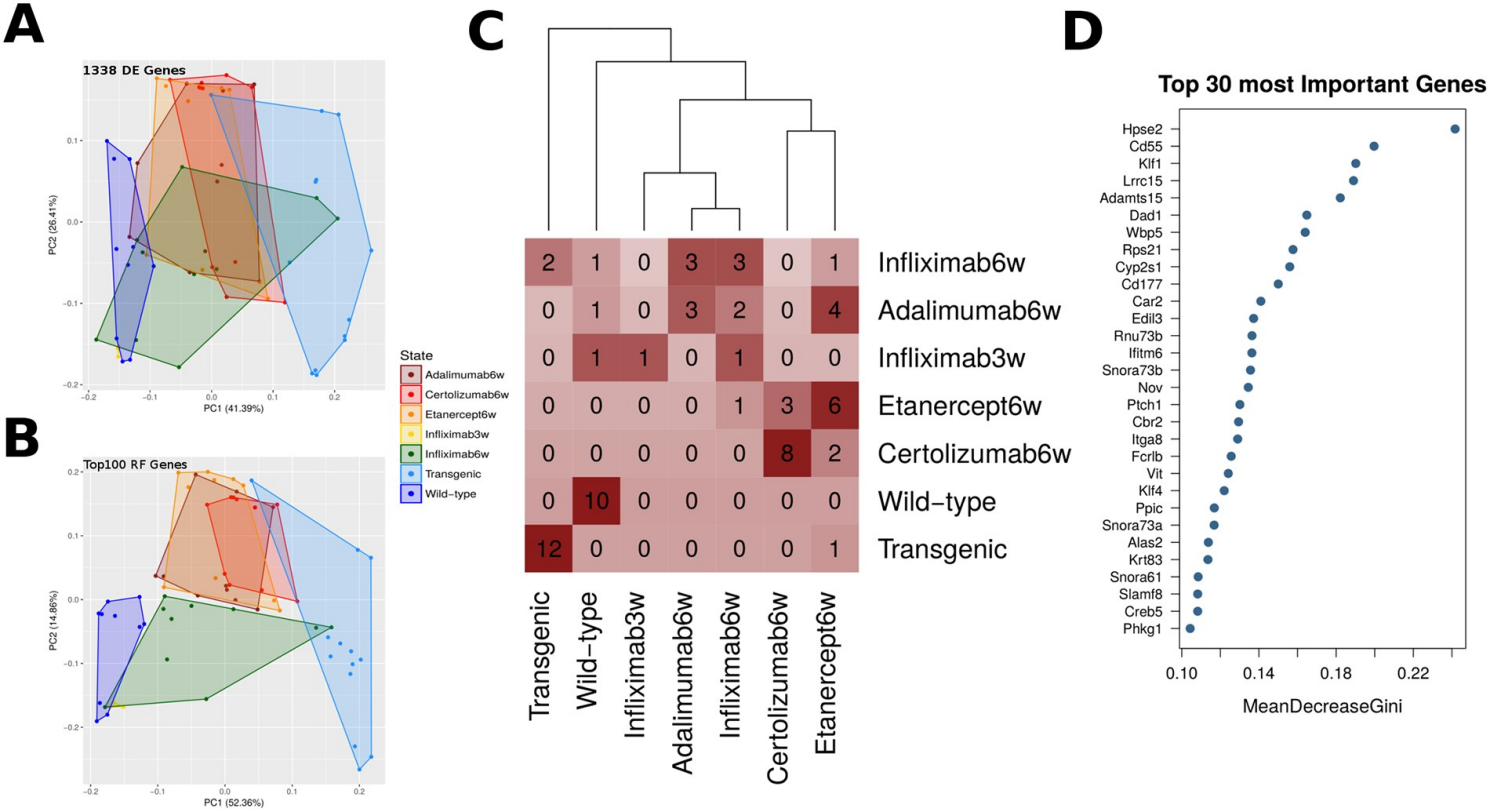
Treatment classification with Random Forests. A. Principal Component Analysis (PCA) plots from all profiles based on a set of 1338 genes that were differentially expressed in at least one condition (disease or treatment) against wild-type (WT) control samples. B. PCA plot on the same profiles based on the top 100 most important genes based on Random Forest Classification. C. Confusion matrix of sample classification according to the best Random Forest Model. Rows correspond to the actual condition of origin. Columns correspond to the condition to which the sample is assigned by the model. Numbers inside cells represent number of samples assigned to each pair. D. Top 30 most important genes according to the best RF model ranked on the basis of mean decrease in Gini coefficient. RF model was trained on all samples.

The results of the Random Forest classification are visualized in Fig 4 first as PCA plots based on the total of 1338 differentially expressed genes (Fig 4A) as opposed to using only the top 100 most important genes based on the Random Forest classification model (Fig 4B). (Notice that given the large number of analyzed features, PCA is used here as an indicative representation of the capacity of the Random Forest model to discriminate between the different conditions). A common characteristic of both PCA analyses is related to a greater variability of transgenic profiles, compared to wild-type and treated samples. Such variability is widely reported in humans and is considered a hallmark of complex diseases [3], however, an even greater number of samples will be required in order to conclude that this variability may be accurately reflected and quantified through our approach. In terms of classification efficiency (Fig 4C) there is a near perfect classification of wild-type and diseased samples, as expected given that the genes under consideration are predominantly differentially expressed between them. Among the various treatments infliximab at both stages of intervention clusters closely to adalimumab. On the other hand, etanercept and certolizumab pegol are classified together in a separate subgroup.

**Fig 5:**
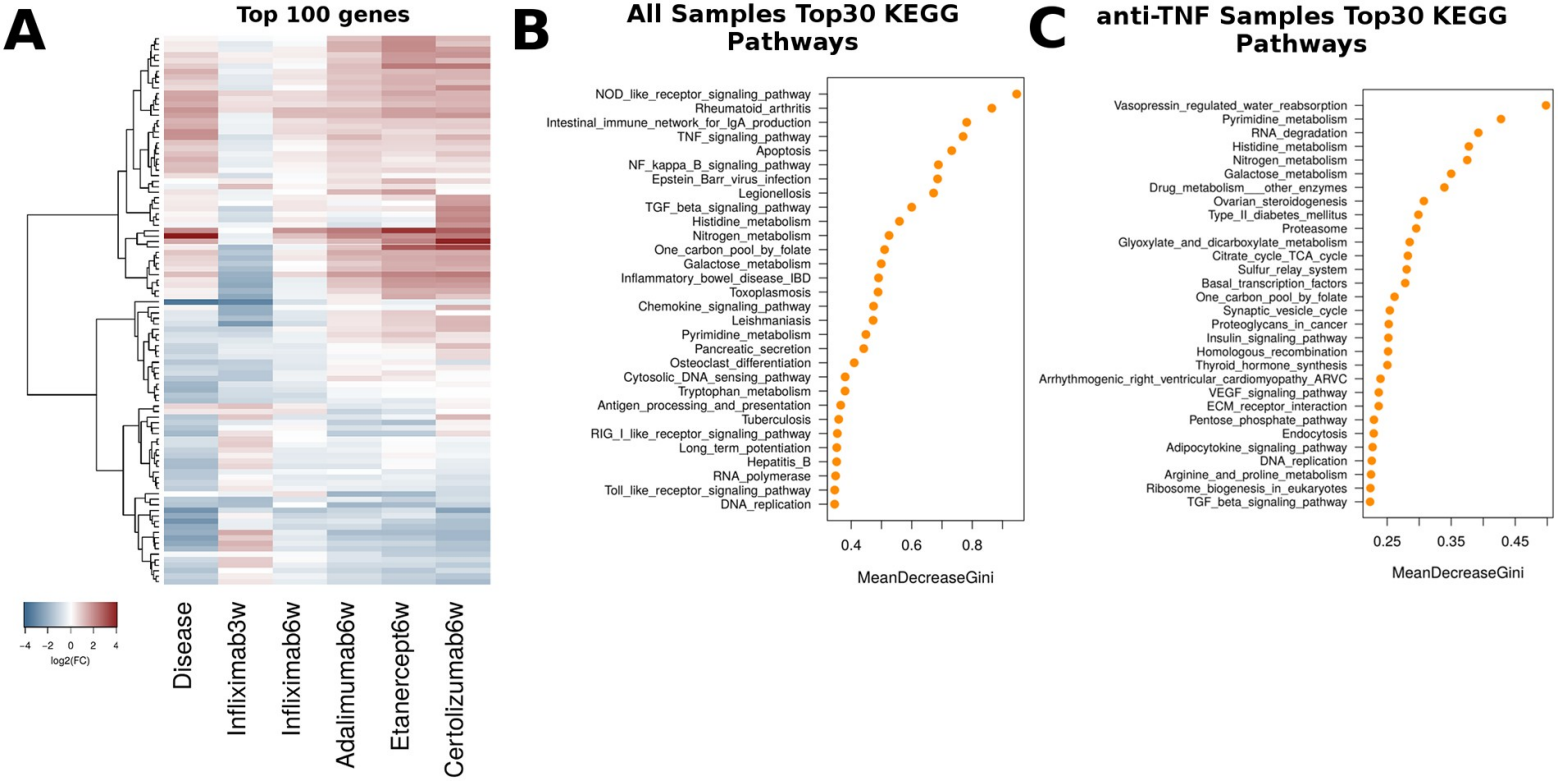
Functional analysis with Random Forests. A. Gene expression levels for the top 100 most important genes used in B. B. Top 30 most important KEGG pathways according to the best RF model when run against pathways instead of genes (see Results for details). RF model was trained on all samples. C. Same as B but for the best RF model when trained exclusively on treatment samples.

When looking closer at the genes predicted by the model to act as the most important classifiers we find a number of genes involved in functions related to the cell adhesion and the extracellular matrix (Fig 4D). The lack of the dominant infectious, immune and inflammatory pathways is expected since the scope of the classification is to define the genes that would better discriminate between all profiles, including the 5 different treatment regimens. Thus, the genes that achieve the best classification are mostly revealing of inter-treatment differences as may be seen in their expression profiles. When looking into gene expression levels of the top 100 most important genes (Fig 5A) one can see a predominance of genes that are under-expressed in diseased animals since it is among them that the greater variability among treatment becomes manifest.

Fig 4A and 4B suggest that the main premise of the random forest classifier is a distinction between the diseased and wild-type states. As this is likely to be obscuring a better discrimination between treatments, we employed an identical classification approach on a restricted set of samples that included only the treated animals. The results of this classification (S6 Fig) show a marked differentiation of infliximab profiles compared to the three other substances, which now show partial but not complete overlaps. The most important genes in the classification are again primarily associated with functions related to the extracellular matrix, development and cell adhesion (S7 and S8 Figs). Inspection of the gene expression levels of the top 300 most important genes shows that classification is based predominantly on two properties: a) the tendency of infliximab at prophylactic intervention to drive a reversal of over-expressed genes and b) the generally limited capacity of certolizumab pegol to restore over-expressed genes (S8 Fig). Together this first level Random Forest analysis suggests that even though significant differences between treatments can be identified, the most important features are somehow lacking descriptive power. That is the genes that best classify the samples were only partially reflecting the system under study. In order to further dissect these differences, we next employed a Random Forest analysis at functional level.

### Random Forest implementation at functional level

We applied an identical Random Forest approach at the functional level, starting from a set of differentially expressed genes, mapping them to the corresponding functional categories and then using the mean expression value as input feature for the model (see Materials and methods). By applying the same strategy as the one described above, we were able to define the most important GO, KEGG Pathway and Transcription Factor (TF) categories in the discrimination of treatment profiles. The results from the best model are summarized in Fig 5B where the top 30 most important KEGG Pathways are shown for the best model applied on the full dataset comprising all samples (including healthy and diseased states). When looking at functional level, both pathways (Fig 5B) and transcription factor features (S9 Fig), accurately reflect the system under study with major inflammatory and immune disease pathways and related transcription factors being among the most important predictive characteristics of the model. This indicates that functional enrichment analyses may confer additional, if not superior insights in the system under study, compared to the more detailed inspection of particular genes.

When classifying treatments in the absence of healthy and diseased profiles, thus having removed strictly disease-associated pathways, a first interesting observation is the role of metabolic pathways, which appear to be dominant both at the level of KEGG Pathways (Fig 5C) and Transcription Factor enrichments (S9 Fig). Nitrogen metabolism in particular, including nucleobase and aminoacid rank among the top most important pathways, while, at the level of TF, immune-response transcription factors such as Nfkb have given their place to metabolic, growth and developmental regulators such as Pparg, Atf1 and Hoxc8 (S9 Fig).

### Two-dimensional treatment profiling analyses

In an attempt to capture a more complete picture of treatment efficiency we calculated mean distances from both wild-type and transgenic samples for each set of profiles. In principle, profiles need to be far from the transgenic but close to the wild-type state, but in the actual data a variety of intermediate profiles may be observed. In order to monitor the range of these responses we have devised a simple two-dimensional approach, that aims to capture, describe and visualize differences between treatment profiles at functional level. The approach is generic which means that it can be readily applied to any gene categorization (see Materials and methods for details) but is, herein, restricted to the level of already discussed GO, KEGG Pathway and TF functional categories.

**Fig 6:**
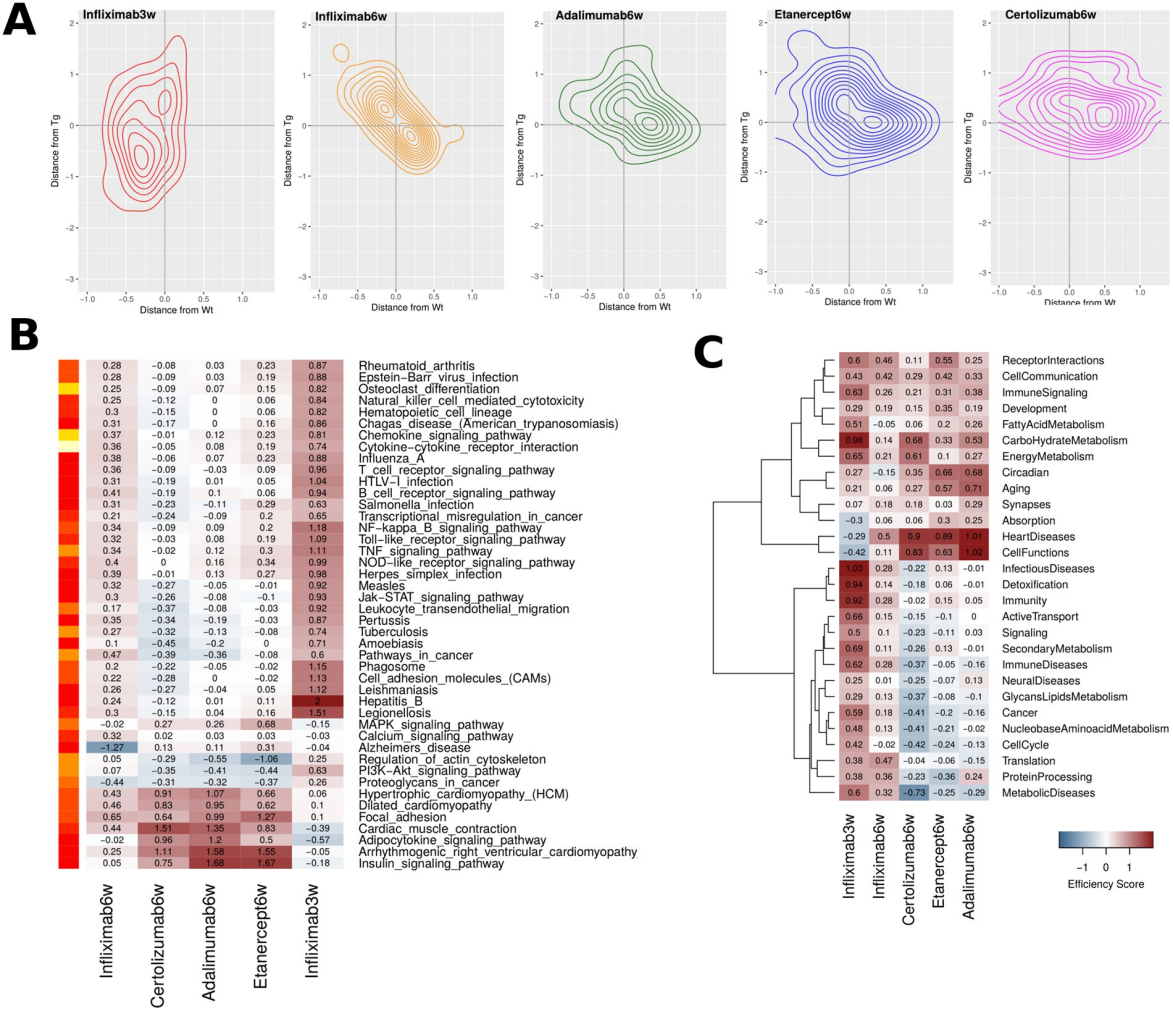
Two-dimensional treatment efficiency profiles. A. Representative two-dimensional contour plots of mean differential expression against wild-type (WT) (horizontal axis) and transgenic (vertical axis). Contours are created with the aggregation of points from 1338 differentially expressed genes that belong to the top 30 most important KEGG pathways (Fig 3D). B. Efficiency scores (see Material and methods for details) of the most important KEGG pathways according to the best RF classification model. Coloured bar on the left corresponds to importance of KEGG pathway in the classification (red: low, white:high). C. Mean efficiency scores for 28 broad KEGG pathway Categories, encompassing more than 200 KEGG pathways and based on all 1338 DE genes. Assignment of pathways to categories is provided as Supplementary Information.

We went on to interpret and visualize these enrichments in the following way: For any given functional category we have calculated a mean distance of expression of each treatment (taking into account only DE genes) against both wild-type and transgenic samples. Then, for a selected list of such functional categories, we plot the distances from both profiles (wild-type and transgenic) in the form of density scatterplots as those shown in Fig 6A. These represent the density of DE genes belonging to the selected functional categories in two dimensions, in which the vertical and horizontal displacements from 0 correspond to the distances from diseased and healthy samples respectively. In this sense, efficient responses are represented by clouds lying around the vertical axis x = 0, with minimal horizontal (low adverse effects) and maximal vertical (high response to disease) values (also see Materials and methods).

Fig 6A shows the combined density plots for the top 30 most important KEGG pathways described by the best model in the Random Forest classification. Infliximab at prophylactic intervention stands out with the most preferable pattern, represented as a cloud along the vertical axis and with a small displacement towards negative values for the distance from wild-type. Differences between the therapeutic interventions may be qualitatively observed in the shape of the density plot. Starting from infiximab and down to certolizumab pegol, the contours flatten out along the horizontal axis pointing to increasing levels of distance from wild-type. All therapeutic interventions share similar patterns with vertical displacements (distance from transgenic) systematically greater than the horizontal ones (distance from wild-type) but with considerable distances from both diseased and healthy states, represented as diagonally diffuse clouds.

This approach may be used for different functional categorizations in order to capture particular characteristics of the response, as may be seen in the case of GO terms or Transcription Factors (S10 and S11 Figs). Again, starting from infliximab the density plots show a gradually increasing displacement along the x-axis, which is representative of insufficient restoration of gene expression to healthy, wild-type levels. Some interesting features of this analysis are related to the persistence of the retinoic acid receptors (S11 Fig) whose targets appear to be invariably under-expressed in transgenic but over-compensated in the treatments. Differences between treatments may be primarily attributed to metabolic regulators such as Pparg and Ppara as well as Atf1, and Atf2, which have been reported as being selectively under-expressed in RA synovial extracts even when compared to osteoarthritis (OA) controls [33].

### Treatment efficiency calculated on the basis of 2D analysis

Two-dimensional density plots may provide a helpful framework for the visualization of the treatment profiles but are not easily quantifiable. Using, the 2D profiles as starting point, we devised a simple measure of response efficiency that aims to capture a combination of a treatment’s potential in a) restoring expression levels that are changed in disease while b) leaving wild-type, healthy levels unaltered. We calculated such an efficiency measure as the log_10_-transformed ratio of the absolute transgenic over the absolute wild-type distance (see Materials and methods for details), for various subsets of functional categories.

**Fig 7:**
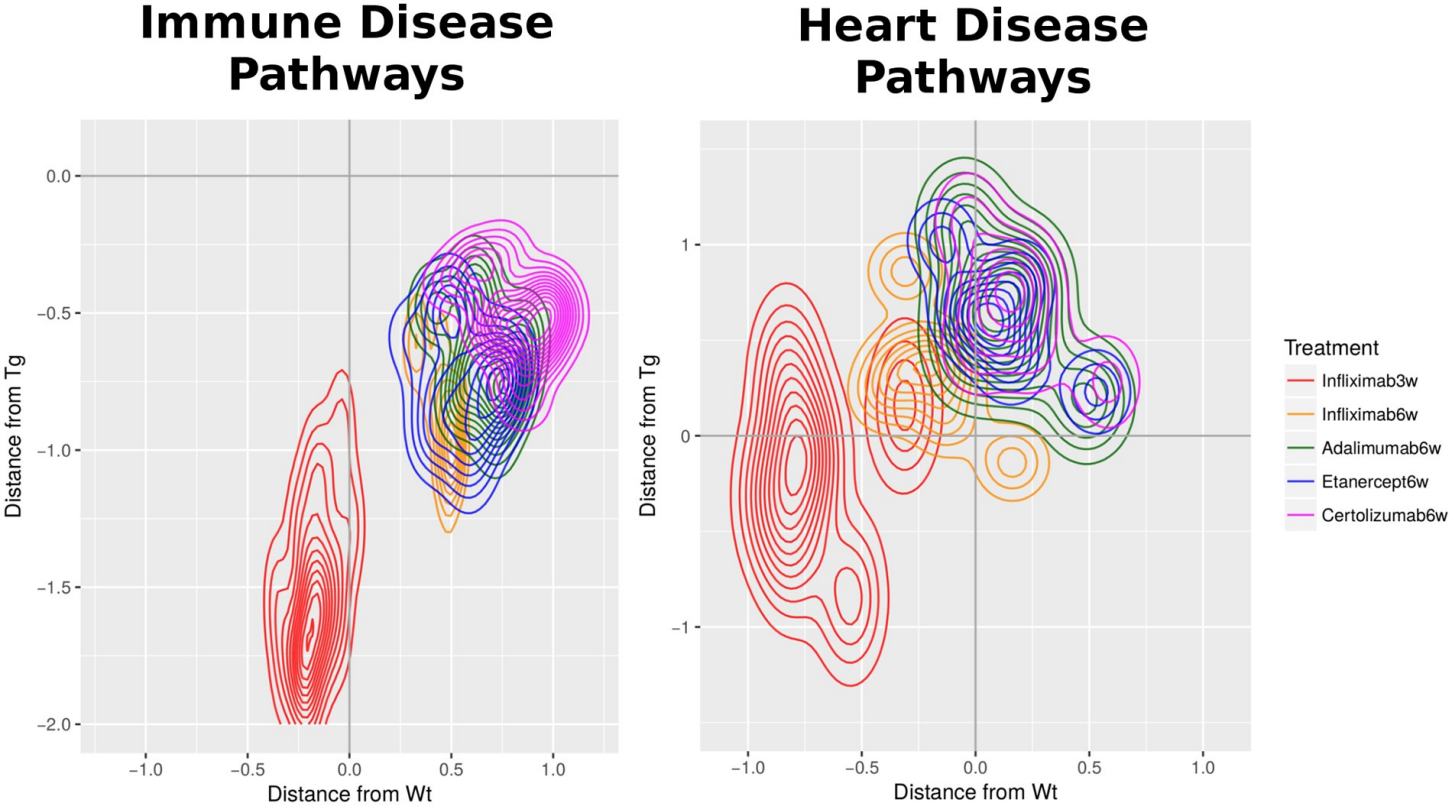
Two-dimensional treatment efficiency profiles. A representative example of composite two-dimensional contour plots for two characteristic pathway categories.

The results for the most important KEGG pathways are shown in Fig 6B, where one may observe particular tendencies of the different responses in great detail. The overall greater efficiency of the prophylactic intervention is again apparent, however there is also a greater consistency in the pattern of infliximab at therapeutic intervention with very few negative scores (which correspond to high frequency of gene expression reversals). Perhaps the most interesting observation from the efficiency analysis comes from the bottom of Fig 6B where one can see increased positive scores for adalimumab, etanercept and, to a lesser extent, certolizumab pegol for a number of functions related to heart disease as well as two metabolic signaling pathways (Adipocytokine and Insulin signaling), for which infliximab shows lower efficiency at both stages of intervention. These results are further supported by more extensive analyses for broader functional sets (Fig 6C) as well as at the level of transcription factor enrichments (S12 Fig). In fact, a recurring observation from various points of our analysis points towards a general pattern for adalimumab and etanercept targeting pathways associated with known RA comorbidities more effectively than infliximab, compared to a less pronounced response against key inflammatory processes which are more readily addressed by the latter (Fig 7).

## Discussion

The development of anti-TNF therapies has been a milestone in the treatment of rheumatoid arthritis. Currently there are 5 different biologics (infliximab, adalimumab, etanercept, golimumab, certolizumab pegol) in the market while novel biologics or biosimilars have also been developed or are under development. Although all of them target the same molecule, they are different in their molecular structures which range from a fusion protein (etanercept), to human (adalimumab) and chimeric (infliximab) mAbs and a PEGylated Fab fragment (certolizumab pegol). Such structural differences may translate to the differences these agents exhibit in antibody-depended cell-mediated cytotoxicity, complement-depended cytotoxicity, capacity to induce apoptosis, ability to neutralize membrane bound TNF or differential inhibition of TNFR1 and TNFR2 signaling [34, 35]. These properties, together with the specific pharmacokinetic and pharmacodynamic profile of each anti-TNF biologic, contribute in shaping their clinical performance profile including their efficacy and safety parameters such as their immunogenicity, as well as the extent to which each specific biologic may affect comorbid pathologies, allergic responses and host defense mechanisms [34, 36–38]. However, comparisons of the different agents exist only in the clinical setting that does not allow head to head or molecular comparisons. With this study we address the need for such comparisons at the mechanistic/molecular level by using an established arthritis model widely used for the preclinical evaluation of anti-TNF therapeutics [12].

To this end we have developed a generalized framework to address differential gene expression in transcriptomic profiles obtained under disease and treatment with 4 different biologics. In the context of RA, we analyze an extended dataset, which consists of a large number of biological replicates and includes both healthy and untreated animals. This enables us to define gene expression changes against both states and thus achieve considerable insight into the way each treatment modulates expression levels. The detail with which the profiles are analyzed allowed us to reveal, previously unreported characteristics related to gene under-expression and off-target responses as well as to pinpoint particular functional attributes that appear, to a great extent, to be treatment-specific. Thus, on one hand, we bring forward hitherto unreported properties of anti-TNF biologics in the context of an established animal model of inflammatory polyarthritis, while, on the other, we describe a concise set of computational analyses for transcriptomic analyses of drug interventions. From the computational analysis point of view, we show that transcriptomics may capture significant differences between anti-TNF treatments that remain largely unobserved at a macroscopic level. Thus, while small differences may be recorded in clinical readouts such as the disease activity scores (which are by definition coarse and subject to noise), more pronounced variability at both quantitative and qualitative levels is revealed through a detailed dissection of gene expression and functional enrichment data, like the one we present in this work. From a computational analysis viewpoint, one particularly interesting aspect is the greater descriptive capacity achieved from the analysis of broader functional terms compared to the “granularity” of genes. As suggested by the last part of our analyses classification at pathway levels provides a more accurate representations of the system under study. These observations are of particular interest when it comes to considering approaches of alignment between animal models and the human condition, as they are suggestive that data integration at hierarchically higher functional levels provides more accurate representations of the conditions under study. Similar systems approaches in human samples, albeit at a smaller scale have shown the increased descriptive power of pathways and modules instead of simple gene signatures [39, 40].

When focusing into the system under study, the response of inflamed synovial tissue to anti-TNF intervention, the results presented in this work point out to a set of functions and terms as immune response, cell communication, cell cycle and signaling already reported for human patients treated with anti-TNF biologics [32, 41] as well as a number of largely overlooked features regarding anti-TNF therapeutic approaches with potentially important implications for the human condition. A first point has to do with the importance of under-expressed genes. Most published works in both humans and animal models focus on over-expressed genes as they are clearly enriched in inflammatory and immune response pathways, targeted by anti-TNF agents. Nevertheless, our data show that more than half of the differentially expressed genes have decreased expression levels compared to healthy controls. These are interestingly enriched in functions that are not directly related to inflammation, such as those associated with heart and muscle development and related diseases as well as secondary metabolic pathways. Such functions are not expected to be directly addressed by anti-TNF action. We find, however, that they are differentially modulated by the substances analyzed, an observation that points to interesting treatment-specific characteristics.

Another interesting aspect regarding under-expressed genes is that they represent a subset of genes and functions for which a prophylactic intervention fails to efficiently restore expression levels. This may be indicative of a time-dependent effect under which an initial wave of over-expression of inflammation genes is followed by a late onset under-expression, which thus escapes the early intervention. The time of intervention is also shown to be important through our analyses of altered genes. Early, prophylactic intervention schemes are unlikely to form part of treatment regimens in humans but are, nonetheless, valuable in the context of animal models as they reveal certain aspects of disease development, that cannot be monitored otherwise. The reduced potential of early intervention to address under-expression may be related to a very different gene expression programme that is characterized by low inflammation. High levels of inflammation in human synovial tissue have been shown to be positive predictors of anti-TNF response [42] and so this could partially explain the shortcomings of the prophylactic treatment.

Last but not least, a number of observations in our work are potentially interesting in the alignment of the mouse with the human condition. The first is the previously overlooked prominence of under-expression of genes related to known RA commorbidities [31] discussed above. A second point is related to the level at which animal models and human patient samples are to be compared. As shown from our random forest analysis, a representative description of the diseased state is better achieved through functional enrichment analysis at pathway level instead of aiming at the definition of gene lists and signatures. In the past we have shown this superiority of pathway level interpretation for the alignment of mouse to human data at both transcriptome and methylome levels [43]. A third point is related to the well-established variability of human patients in terms of both disease severity and response to therapy [13, 16–17]. Even though the number of samples in our study is limited, such variability is replicated with disease samples showing broader expression patterns compared to wild-type controls, as is evident in both the PCA analyses and the distributions of expression values. Perhaps the most interesting implication of our study may be that the significant quantitative and qualitative differences we detect between different anti-TNF agents are underlying the well-studied variations in patient response.

In all, our approach involving head to head comparisons of different anti-TNF biologics aligns to the limited human data and enables us to capture subtle, or more profound differences between anti-TNF agents and to quantify them through an innovative scheme of efficiency scores. We herein show that transcriptomic analyses represent a valuable means for the study of disease mechanisms and the intricate modes of action of specific treatments. The developed computational pipeline may be easily modified and extended to accommodate comparative analyses of drug similarity or small molecule inhibitor efficacy by quantitatively highlighting treatment-dependent discriminatory characteristics. Moreover, such pipelines might be a tool to support the preferential use of a particular agent or class of agents in specific clinical pathology niches thus driving personalized medicine approaches.

## Materials and methods

### Mice and treatments

WT and human TNF transgenic mice (Tg197) [12] were bred and maintained in a mixed CBA×C57BL/6J genetic background in the animal facilities of Biomedcode Hellas S.A. under specific pathogen-free conditions. Animals were housed in standard plastic cages with wood chip bedding. The animal facility was under an inverted 12:12-h light/dark cycle at a constant temperature of 22 ± 2 °C and relative humidity of approximately 60%. Food pellets and filtered water were provided *ad libitum*. Experiments were approved by the BSRC Al. Fleming Institutional Committee of Protocol Evaluation in conjunction with the Veterinary Service Management of the Hellenic Republic Prefecture of Attika according to all current European and national legislation and were performed in accordance with relevant guidelines and regulations.

Animals were treated in a therapeutic regimen from week 6 of age or in a prophylactic regimen from week 3 of age. Groups of mice of the same age (gender balanced) received either saline, infliximab (Remicade, Janssen Biotech), certolizumab pegol (Cimzia, UCB) or adalimumab (Humira, Abbvie) administered at 10 mg/kg intraperitoneally twice weekly or etanercept (Enbrel, Pfizer) administered subcutaneously at 30 mg/kg thrice weekly. At the end of the treatment period all mice were sacrificed and hind limbs were flash frozen.

### Performance of experiment. Gene expression arrays (AffyChip)

Total RNA was isolated using Trizol reagent from frozen tissues of wild-type (healthy), huTNFTg (diseased) and huTNFTg mice that were treated with one of the 4 different agents in therapeutic or prophylactic regimen as shown in S1 Table. All therapeutic interventions were carried out in 10 biological replicates, while infliximab prophylactic was carried out in 3. Wild-type and transgenic mice were analyzed in replicates of 10 and 13 respectively for a total sum of 66 profiles.

All samples were hybridized on the Affymetrix GeneChip Mouse Gene 2.0 ST array. Data analysis was performed using Transcriptome Analysis Console (TAC 4.0) Software, Applied Biosystems. CEL files were quantile-normalized with RMA. Log-transformed expression measurements were then converted to gene space by calculating mean probeset values referring to the same genes. This was done in order to minimize the complexity of alternative transcript abundance, which we considered, at this level, to be minimal. Only values from genes that were measured in all samples were finally included in the dataset, which consisted of 18704 common measured genes in all 66 samples (S1 Data).

### Differential expression analysis

Differential expression analysis is usually calculated against a single background condition that corresponds to a baseline control. We took advantage of our experimental setup and calculated differential expression in the treated samples against both wild-type samples, to quantify gene expression changes versus the native, healthy state, and untreated huTNFTg transgenic samples, to assess changes relative the diseased state. This is a considerable advantage of our approach compared to human studies where the lack of healthy controls often undermines a series of comparisons. Differential expression was calculated as log_2_Fold-Change (log_2_FC) values from a one-way ANOVA followed by Dunnett’s test for multiple comparisons using either Wild-type (WT) or Transgenic (TG) samples as control condition. The differential expression analysis was implemented in R and is included in S1 Code. Standard thresholds of |log_2_FC|≥1 and an adjusted p-value≤0.05 were applied for the definition of differentially expressed genes.

### Clustering of differentially expressed genes

A combination of clustering methods was employed in the clustering of genes and functional enrichments, as well as in the clustering of treatment efficiency scores (see below). Genes and conditions were clustered with agglomerative hierarchical clustering using Ward’s minimum variance criterion [18]. The optimal number of clusters was defined in all cases, on the basis of a Silhouette consistency analysis [19].

### Functional analysis

Functional analysis was performed with the use of gProfileR [20], through its R package implementation and separately at the levels of GO terms (BP: Biological Process, MF: Molecular Function, CC: Cellular Component), KEGG pathways and Transcription Factor targets. Transcriptional regulator targets were incorporated through the repositories of RNEA [21].

### Profile similarity calculation

Profile similarity/distance was assessed in the form of the mean absolute difference of gene expression as log_2_FC. Thus, for a given number of *N* genes the distance between two profiles (*P*_*1*_, *P*_*2*_) is defined as:

$$d\left(P_{1,}P_{2}\right)=\frac{\sum_{\text{i}=1}^{N}\left|{\text{log}}_{2}\text{FC}\left(P_{1}\right)g_{i}-{\text{log}}_{2}\text{FC}\left(P_{2}\right)g_{i}\right|}{N}$$ (1)

Profile distance from wild-type samples was used as a proxy for treatment efficiency, calculated for the entire set of differentially expressed genes, as well as for various subsets defined through the clustering approaches described above.

### Gene intersection analysis

Comparison of multiple gene lists leads to complex intersection patterns. We used UpSetR [22], an R implementation of the UpSet technique [23] to analyze the intersection between various gene lists. UpSetR produces visualizations of complex set intersections in a matrix-based layout, while also providing information on the original set sizes.

### Random Forest classification

We employed a Random Forest (RF) [24] classification strategy to define subsets of genes and functional categories that best discriminate healthy from diseased profiles as well between various treatments. Random Forests were implemented through the randomForest R package. Starting from the complete dataset we used a 70/30% split for training and test sets respectively and built 1000 RF models with 500 trees each, using 10 variables at each split. From the 1000 RF models we chose the one with the lowest out-of-bag error rate and obtained the variables with the greatest importance on the basis of the higher Mean Gini Decrease (MGD). Arbitrary thresholds for the top most important predictors were applied depending on the downstream analyses (e.g. 30 or 50 for representation reasons, 100 for Principal Component Analysis).

Random Forest classification was implemented in two different datasets. One with all samples, that included wild-type and untreated transgenic samples and one that excluded them, focusing only on the treatment profiles. We used the first to observe broader differences between healthy and diseased states and the second to provide us with a more detailed view of the treatment-specific characteristics.

### Two-dimensional distance analysis and treatment efficiency

Two-dimensional analysis of gene expression was calculated as a combination of differential expression values against wild-type (healthy) and transgenic (diseased) mice profiles. The two-dimensional approach was aggregated at functional level. For each functional category we obtained the DE genes that belonged to that category and then, for each treatment, we calculated the mean log_2_FC value for this subset of genes versus both wild-type and transgenic samples. This pair of values was then used in two-dimensional representations of treatment profiles and in the calculation of treatment efficiency scores. The two-dimensional density plots represent the landscape of response of a given treatment for a certain subset of genes or functions. They are formed through the aggregation of pairs of distances (from wild-type and transgenic) for functional categories specified by the experimenter or derived from previous analyses (such as the classification schemes described above). Each treatment landscape is thus visualized as a contour cloud, the shape and size of which is representative of its efficiency. Displacement along the transgenic (vertical) axis corresponds to desirable distances from the diseased state, while displacement from the wild-type (horizontal) axis is typical of undesirable effects that place the treatment at a distance from the healthy condition.

The relative amplitudes of this data cloud may be quantified in the form of efficiency scores. These are calculated as the log_10_-based values of the ratios of mean absolute gene expression values of DE against transgenic (TG) over wild-type (WT) profiles, according to the following formula:

$$E\left(\text{P},\text{f}\right)={\text{log}}_{10}\frac{\left|\bar{{\text{log}}_{2}\text{FC}\left(\text{Tg}\right)g_{i}}\right|}{\left|\bar{{\text{log}}_{2}\text{FC}\left(\text{Wt}\right)g_{i}}\right|}$$ (2)

where *E(P*,*f)* is the efficiency score of profile (treatment) *P* for the functional category *f*, which contains *N* differentially expressed genes. In (2), *g*_*i*_ corresponds to a set of *N* genes belonging to a given category, over which the mean absolute log-fold-change is calculated. High efficiency scores are thus obtained for functional groups with genes having large absolute changes when compared against transgenic, and low when compared to wild-type profiles.

### Data and code availability

All analyses were performed in the R environment with the combination of custom scripts and available libraries. Annotated code is provided in a R Mardown file as S1 Code and the processed data files, required for the full replication of our analysis are provided in one compressed folder as S1 Data.

### Ethics approval and consent to participate

Animal experiments were approved by the Veterinary Service Management of the Hellenic Republic Prefecture of eastern Attika (Approval license Protocol No. 2478, 17/01/2011).

### Availability of data and material

All processed data generated or analysed during this study are included in this published article. Processed files are provided in a single zipped folder (S1 Data). The code for the analysis is also provided as a R Markdown file (S1 Code).

## Supporting information

S1 Fig Gene expression changes in different clusters of disease-associated genes. Dotted vertical line at 0 represents wild-type levels. Names of clusters defined by observation of the expression patterns.
(TIFF)

S2 Fig Distribution of DE genes across treatments based on gene expression changes against transgenic and wild-type profiles. (TIFF)

S3 Fig Differential gene expression values for 1064 genes including DE unchanged in disease. Genes are divided in 10 clusters according to a Silhouette consistency test.
(TIFF)

S4 Fig Distance from WT for 1064 genes including DE unchanged in disease clustered in 10 clusters. (TIFF)

S5 Fig A. Top enriched functional analysis terms for genes restored, not restored and altered by Infliximab. Restored genes (green) are ~90% common, not restored were therapeutic-specific and altered genes were prophylactic-specific. B. Top enriched functional analysis terms for genes restored, not restored and altered by Adalimumab. C. Top enriched functional analysis terms for genes restored, not restored and altered by Etanercept. D. Top enriched functional analysis terms for genes restored, not restored and altered by Certolizumab pegol.
(TIFF)

S6 Fig PCA plots of profiles of treatment only samples based on all 1338 DE genes (left) and the top 100 most important genes according to the best RF model (right). (TIFF)

S7 Fig Top 30 most important genes according to the best RF model trained exclusively on treatment samples. (TIFF)

S8 Fig Differential gene expression levels for the top 300 most important genes according to the best RF model trained exclusively on treatment samples (left) and Top enriched functional terms for the same 300 genes (right). (TIFF)

S9 Fig Top 30 most important transcription factors according to the best RF model trained on a) all samples (wild-type, diseased, treatments) (left) and b) only on treatment samples (right). (TIFF)

S10 Fig Two-dimensional mean contour plots for the top 50 most important GO functional terms for all five treatment regimens. (TIFF)

S11 Fig Two-dimensional mean contour plots for the top 30 most important Transcription Factors for all five treatment regimens. (TIFF)

S12 Fig Efficiency Score heatmaps for the top 30 most important Transcription Factors for all five treatment regimens. (TIFF)

S1 Table Samples used in this study. (DOCX)

S1 Code A detailed description of the analyses conducted in R in a single R Markdown report. (RMD)

S1 Data A compressed directory containing all the necessary files for the replication of the conducted analyses including a) normalized gene expression values b) log(FC) values c) annotated gene expression values according to functional categories. (ZIP)
