# Supplementary figures and images for: An integrative transcriptome analysis framework for drug efficacy and similarity reveals drug-specific signatures of anti-TNF treatment in a mouse model of inflammatory polyarthritis
Source: PLoS Comput Biol. 2019 May 9;15(5):e1006933. doi: 10.1371/journal.pcbi.1006933 (PMC6508611; doi:10.1371/journal.pcbi.1006933)

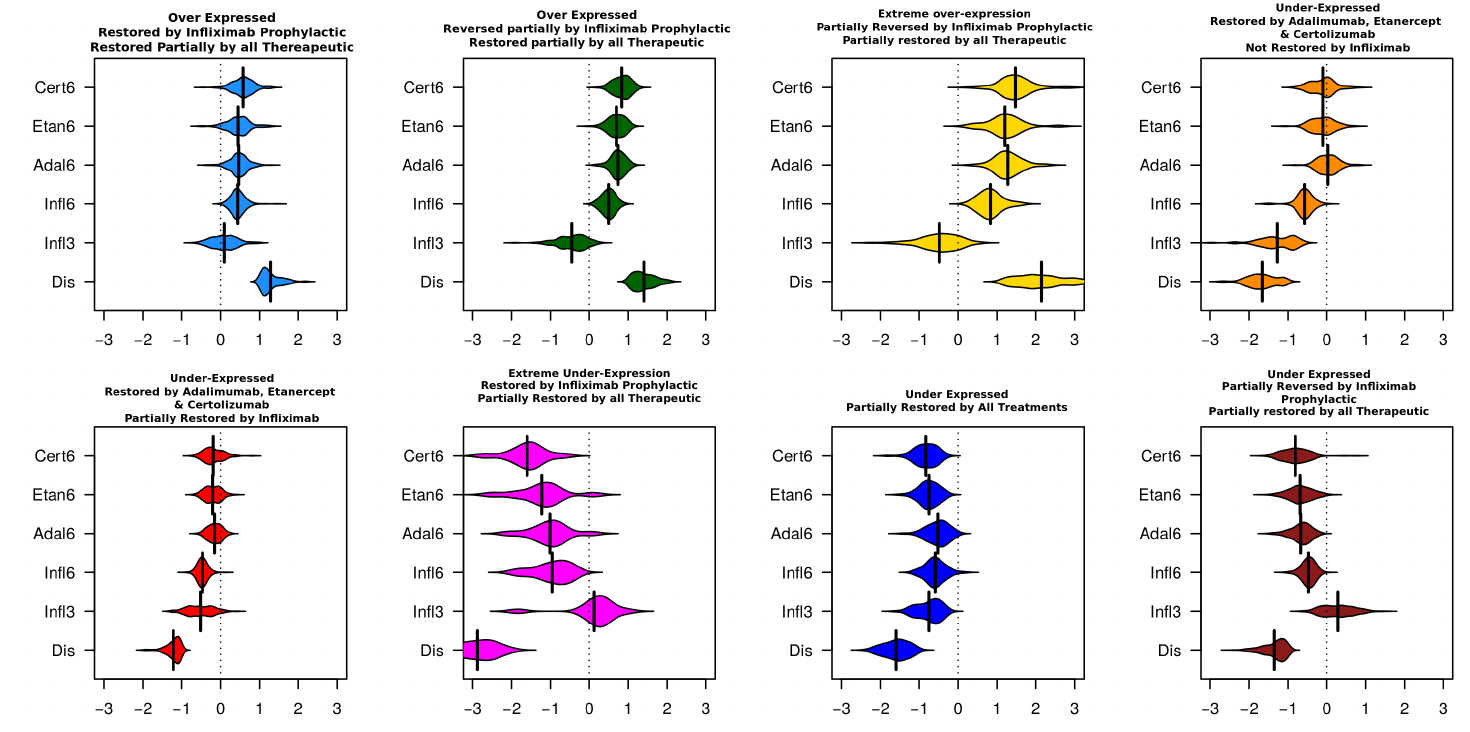

Supplement: S1 Fig — Dotted vertical line at 0 represents wild-type levels. Names of clusters defined by observation of the expression patterns. (TIFF) [file pcbi.1006933.s001.tiff]

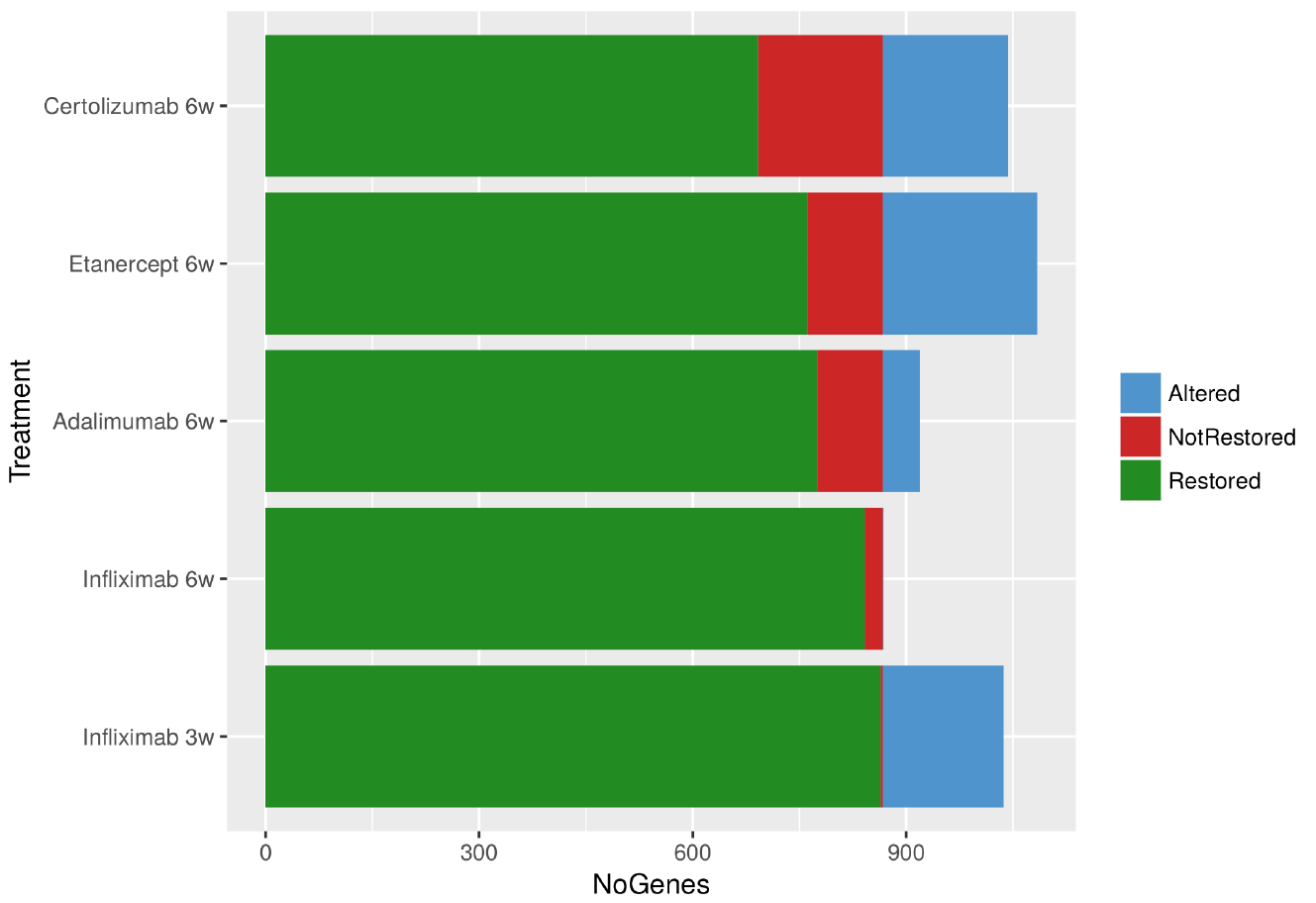

Supplement: S2 Fig — (TIFF) [file pcbi.1006933.s002.tiff]

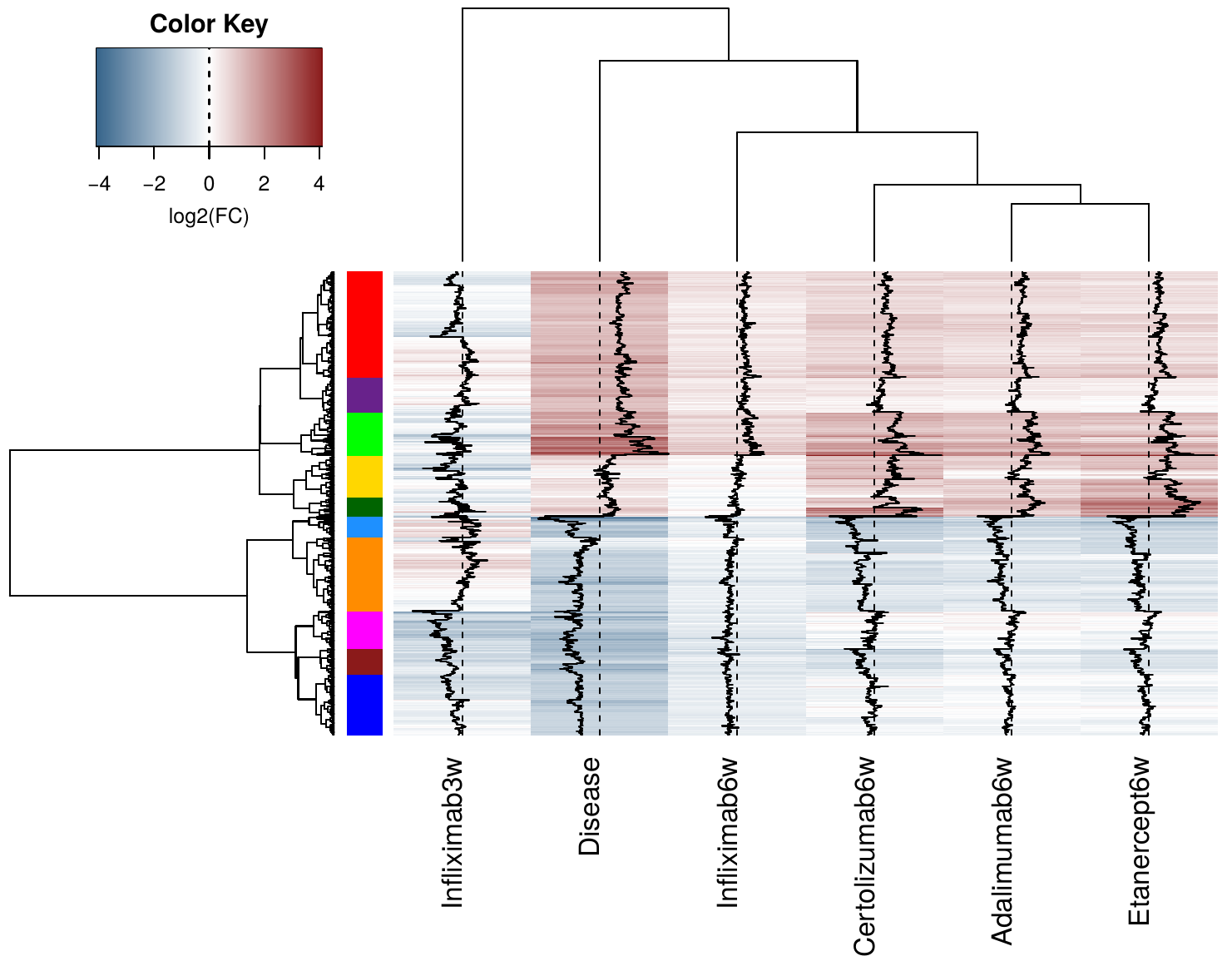

Supplement: S3 Fig — Genes are divided in 10 clusters according to a Silhouette consistency test. (TIFF) [file pcbi.1006933.s003.tiff]

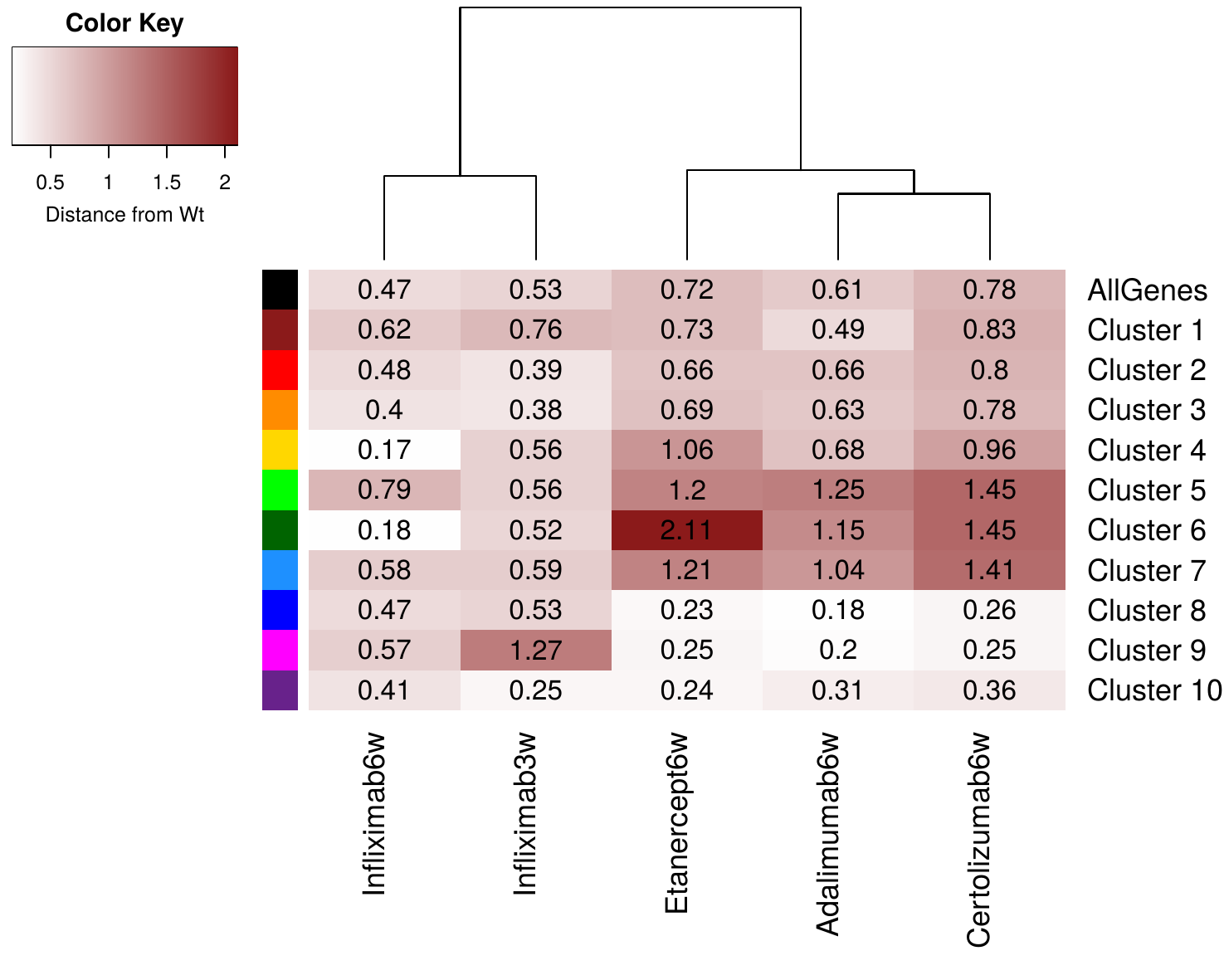

Supplement: S4 Fig — (TIFF) [file pcbi.1006933.s004.tiff]

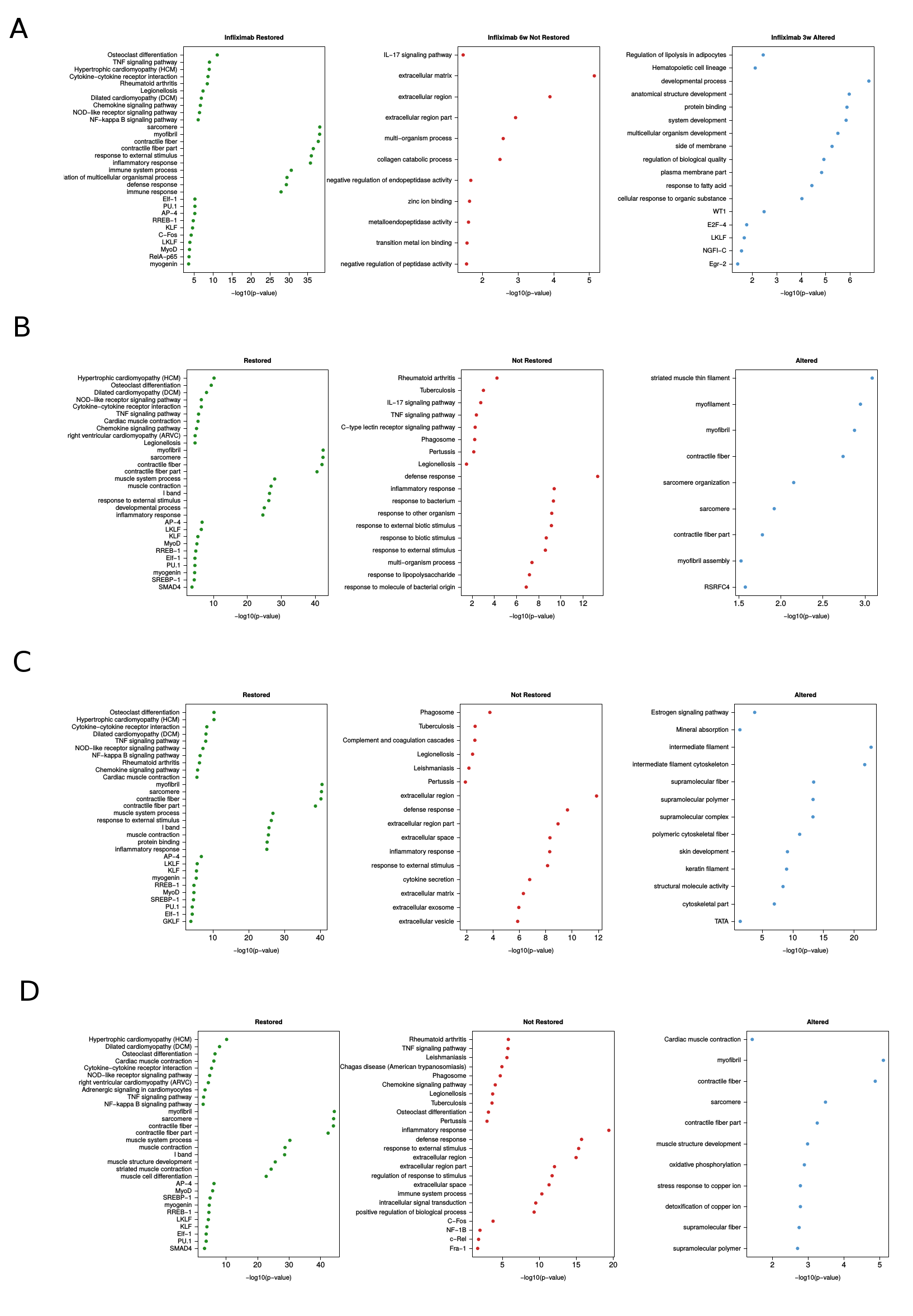

Supplement: S5 Fig — A. Top enriched functional analysis terms for genes restored, not restored and altered by Infliximab. Restored genes (green) are ~90% common, not restored were therapeutic-specific and altered genes were prophylactic-specific. B. Top enriched functional analysis terms for genes restored, not restored and altered by Adalimumab. C. Top enriched functional analysis terms for genes restored, not restored and altered by Etanercept. D. Top enriched functional analysis terms for genes restored, not restored and altered by Certolizumab pegol. (TIFF) [file pcbi.1006933.s005.tiff]

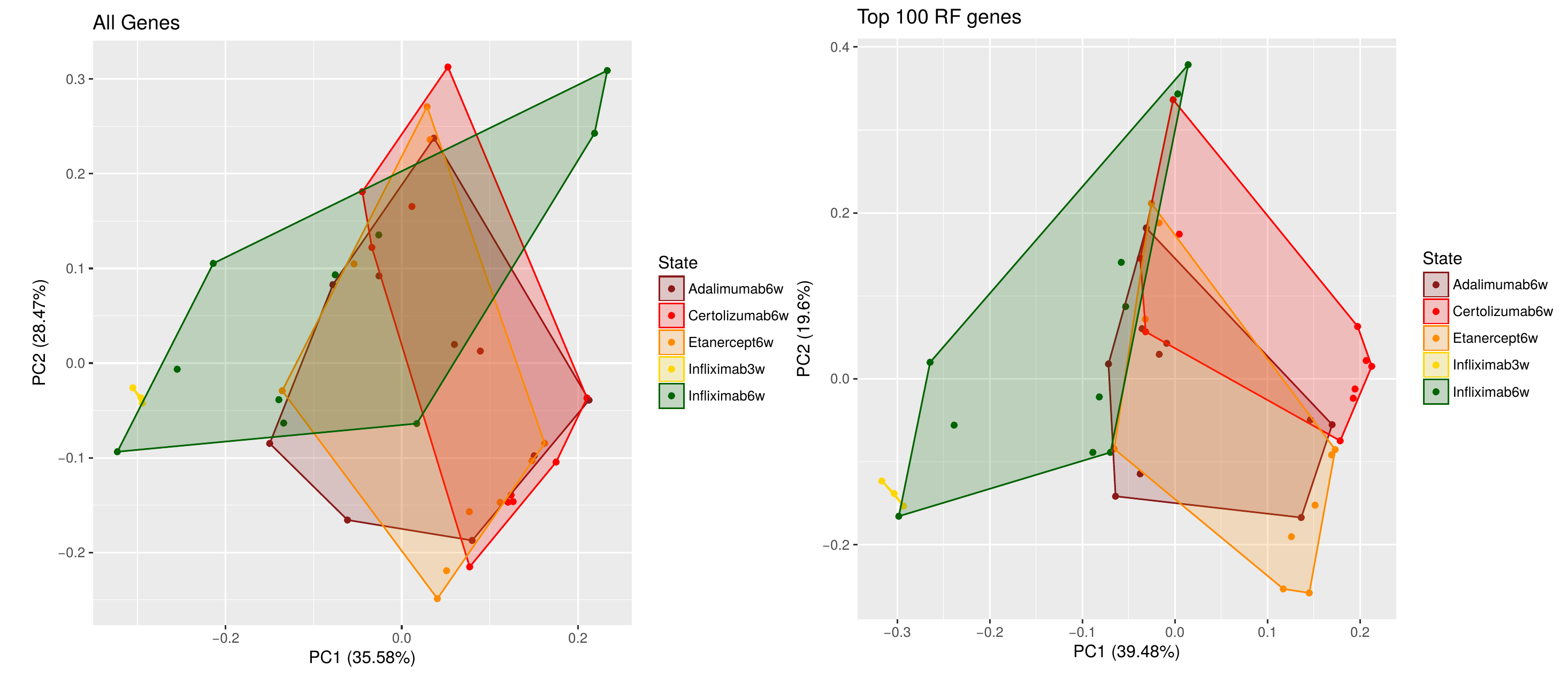

Supplement: S6 Fig — (TIFF) [file pcbi.1006933.s006.tiff]

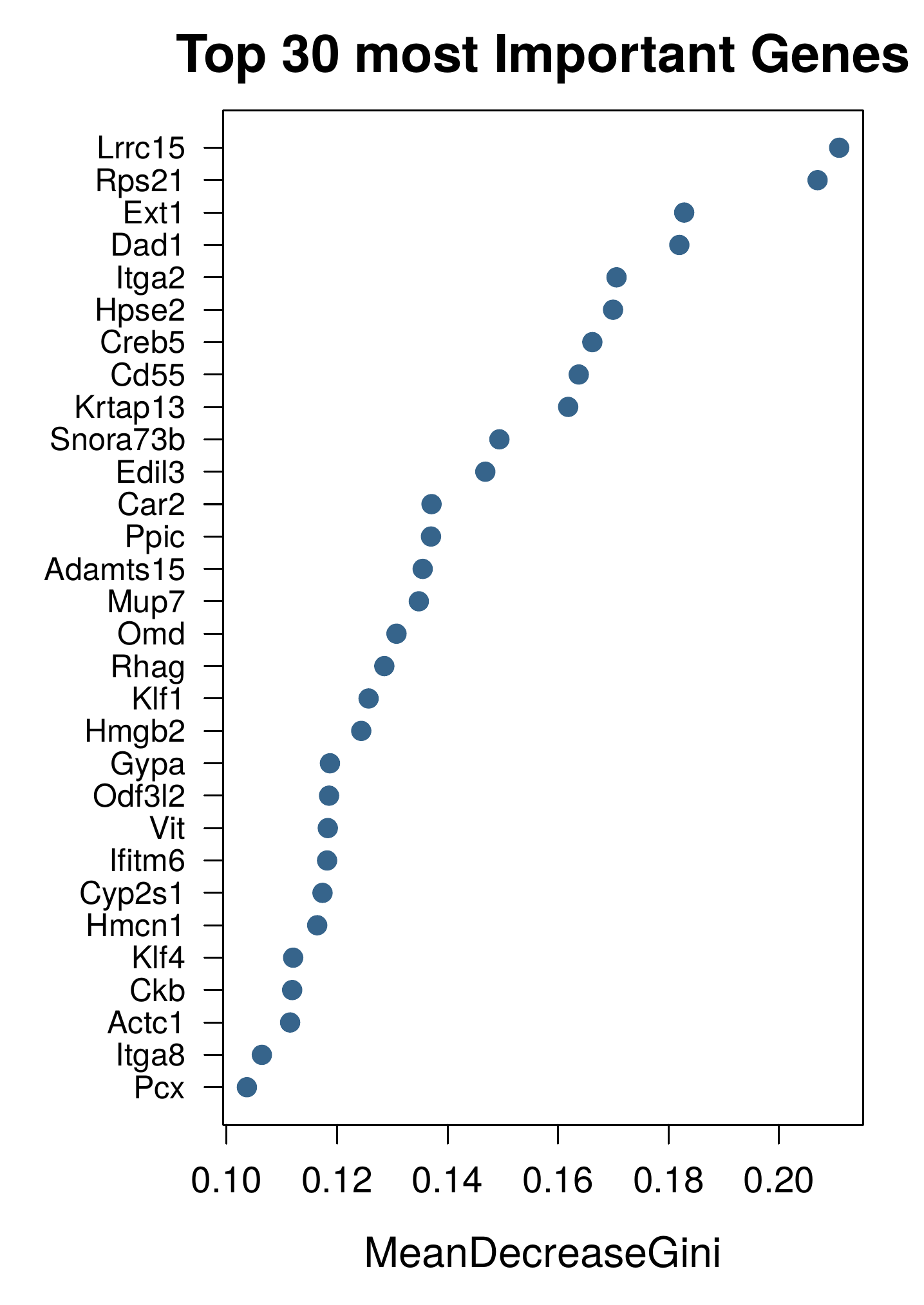

Supplement: S7 Fig — (TIFF) [file pcbi.1006933.s007.tiff]

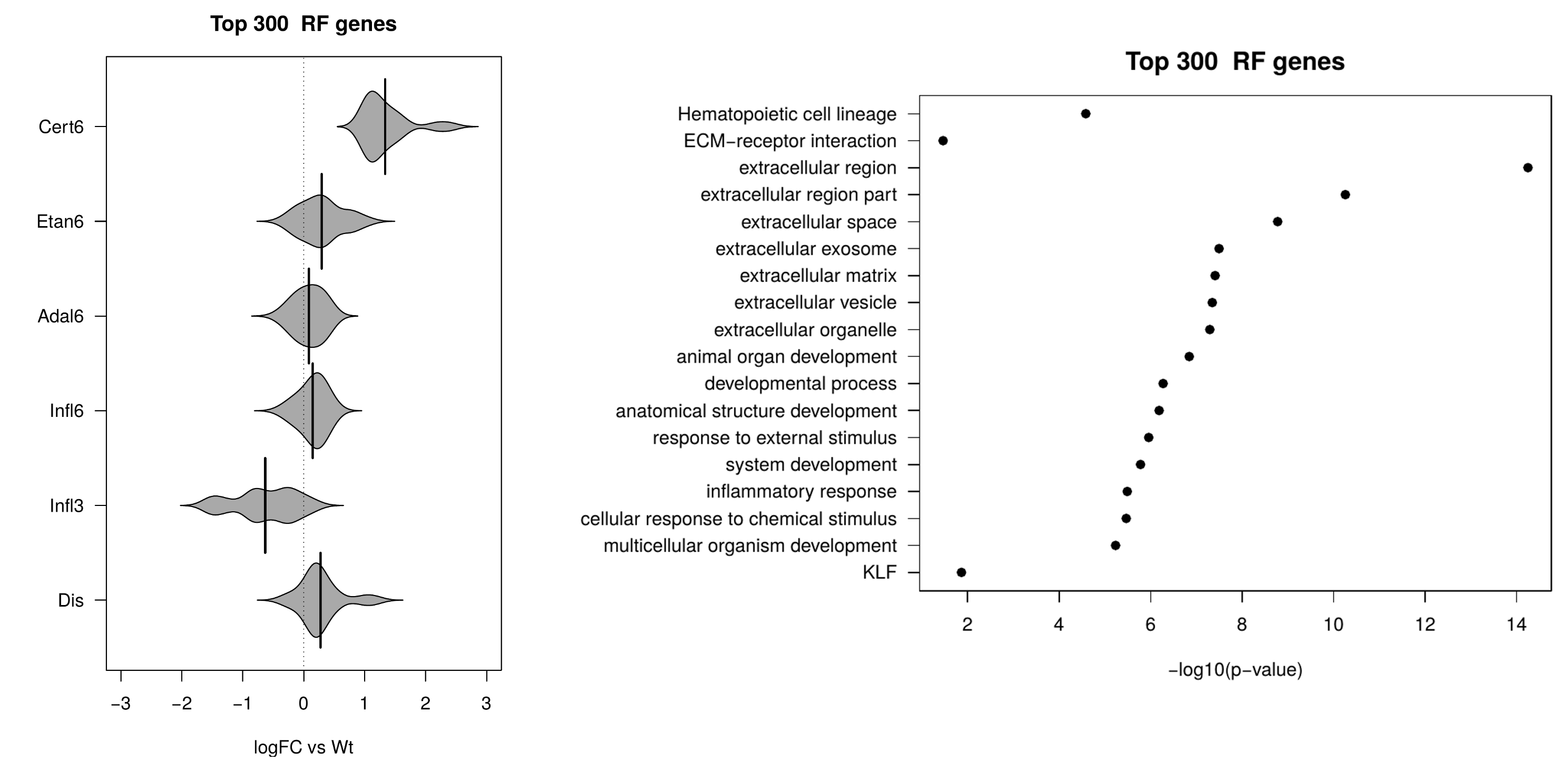

Supplement: S8 Fig — (TIFF) [file pcbi.1006933.s008.tiff]

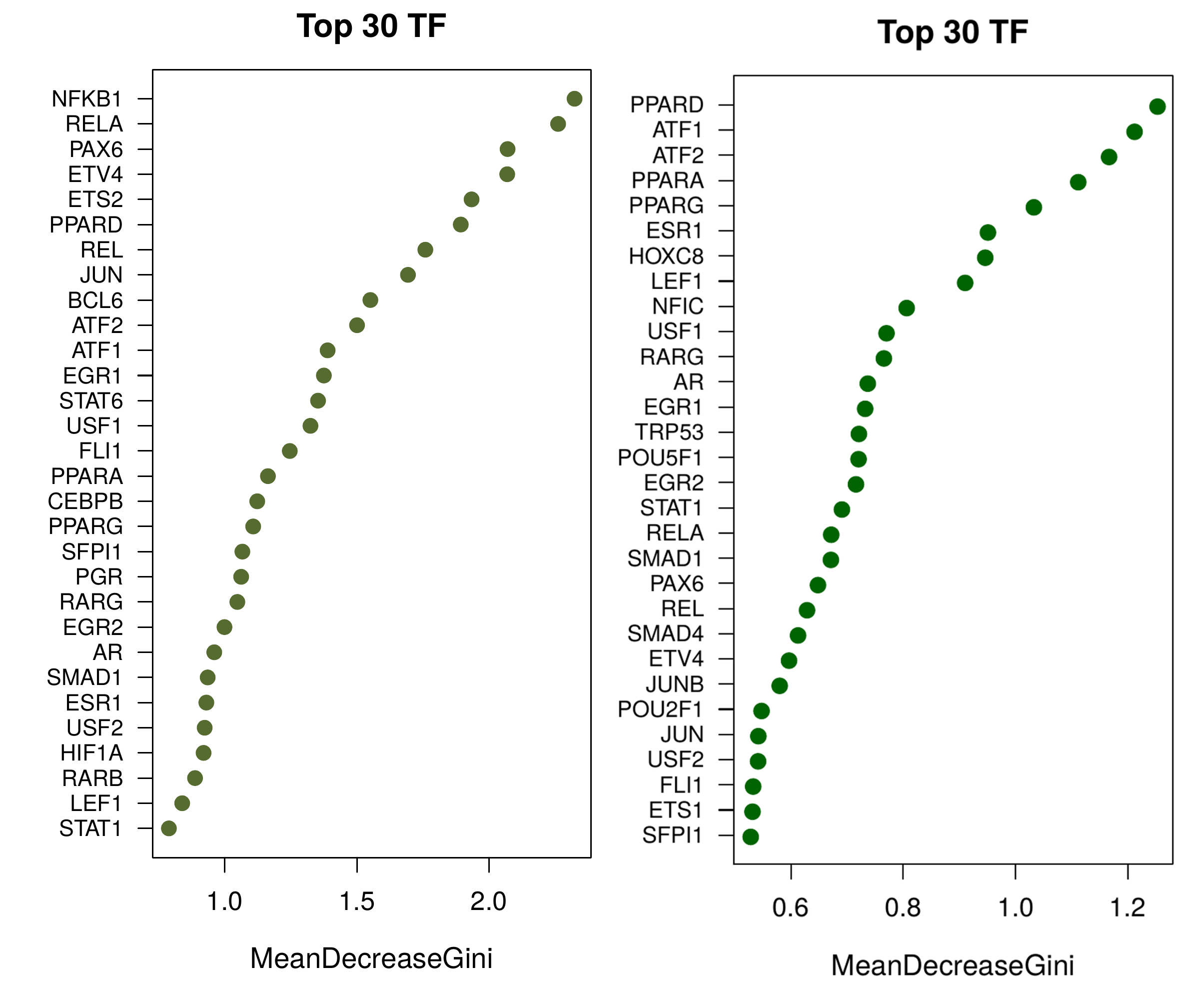

Supplement: S9 Fig — (TIFF) [file pcbi.1006933.s009.tiff]

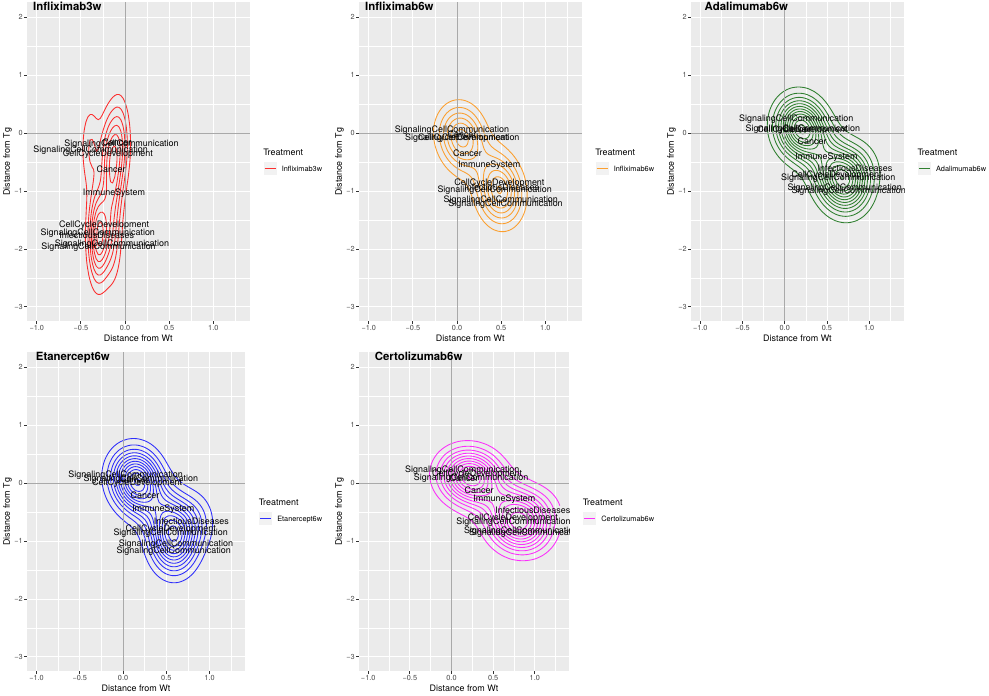

Supplement: S10 Fig — (TIFF) [file pcbi.1006933.s010.tiff]

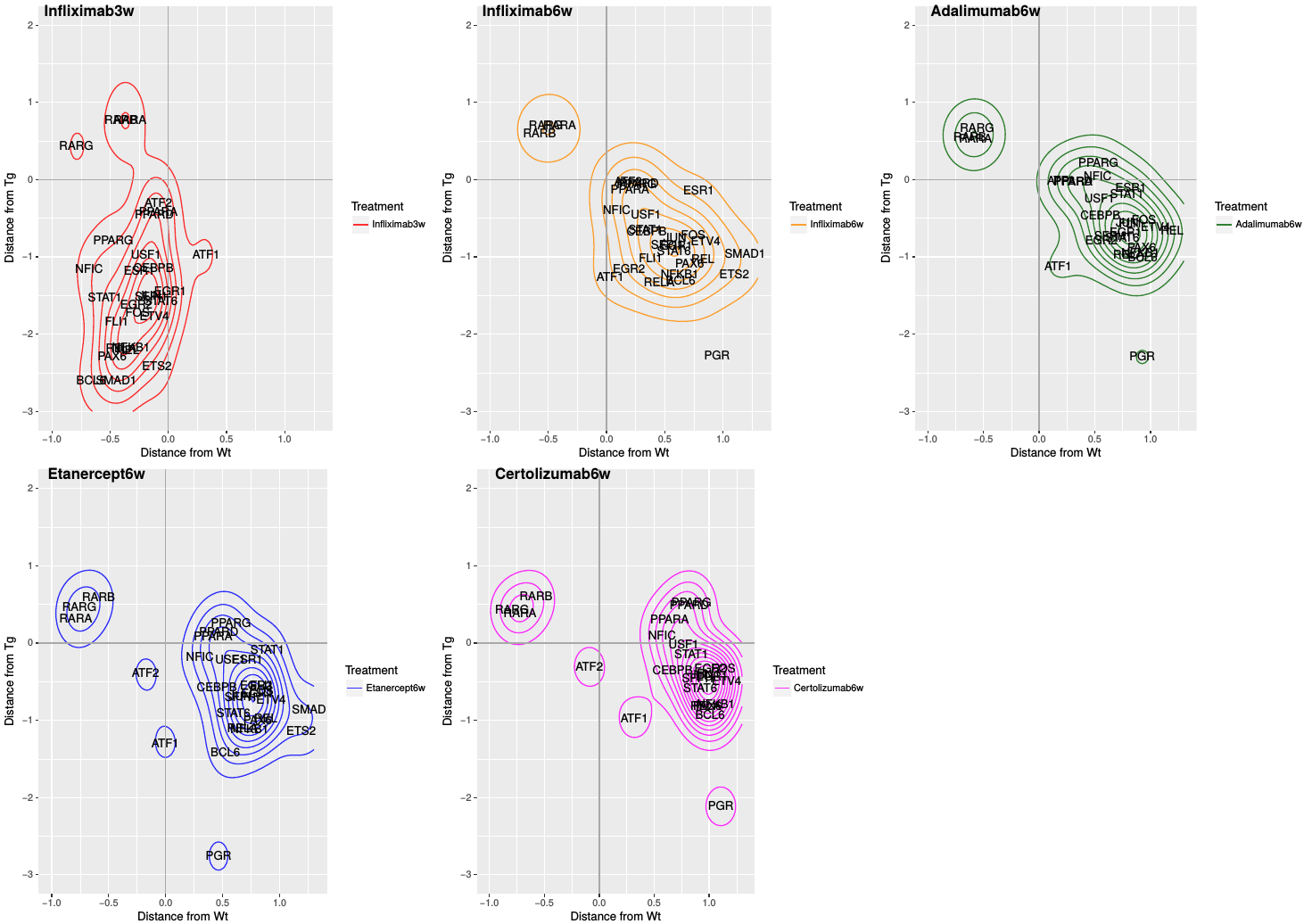

Supplement: S11 Fig — (TIFF) [file pcbi.1006933.s011.tiff]

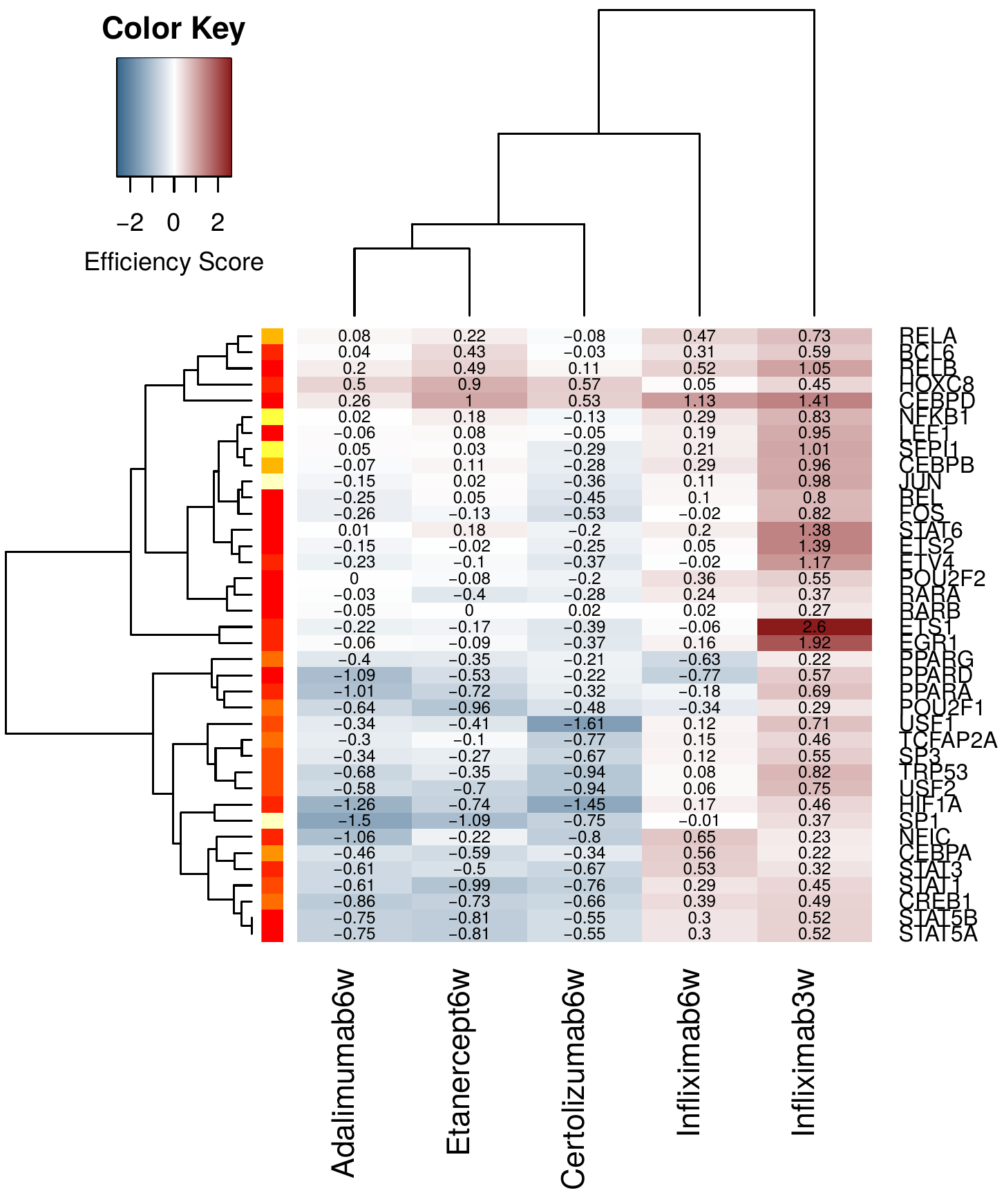

Supplement: S12 Fig — (TIFF) [file pcbi.1006933.s012.tiff]
