# Supplementary material for: An integrative transcriptome analysis framework for drug efficacy and similarity reveals drug-specific signatures of anti-TNF treatment in a mouse model of inflammatory polyarthritis
Source: PLoS Comput Biol. 2019 May 9;15(5):e1006933. doi: 10.1371/journal.pcbi.1006933 (PMC6508611; doi:10.1371/journal.pcbi.1006933)
Supplement: S1 Table — (DOCX) [file pcbi.1006933.s013.docx]

| **Sample** | **Number of Biological Replicates** |
| --- | --- |
| Wild-type (healthy) | 10 |
| hTNFTg(diseased) | 13 |
| hTNFTg treated with **infliximab** from 3 weeks of age (prophylactic intervention) | 3 |
| hTNFTg treated with **infliximab** from 6 weeks of age (therapeutic intervention) | 10 |
| hTNFTg treated with **adalimumab** from 6 weeks of age (therapeutic intervention) | 10 |
| hTNFTg treated with **etanercept** from 6 weeks of age (therapeutic intervention) | 10 |
| hTNFTg treated with **certolizumab pegol** from 6 weeks of age (therapeutic intervention) | 10 |
